# Identification and External Validation of a Transcription Factor-Related Prognostic Signature in Pediatric Neuroblastoma

**DOI:** 10.1155/2021/1370451

**Published:** 2021-12-28

**Authors:** Rujia Wang, Qian Wang

**Affiliations:** Department of Pediatrics, Shanghai General Hospital, Shanghai Jiao Tong University School of Medicine, Shanghai, China

## Abstract

**Background:**

Neuroblastoma is a common solid tumor originating from the sympathetic nervous system, commonly found in children, and it is one of the leading causes of tumor-related deaths in children. In addition to pathological features, molecular-level features, such as how much gene expression is present and the mutational profile, may provide useful information for the precise treatment of neuroblastoma. Transcription factors (TFs) play an important regulatory role in all aspects of cellular life activities. But there are currently no studies on transcription factor-based biomarkers of neuroblastoma prognosis, and this study is much needed.

**Methods:**

We downloaded RNA transcriptome data and clinical data from the TARGET database to construct a prognostic model. The prognostic model was constructed by using univariate Cox analysis, LASSO, and multivariate Cox regression. We divided the patients into low-risk and high-risk groups using the median value of the risk score as the cut-off. Then, we validated the prognostic model with the dataset GSE49710.

**Results:**

We constructed a prognostic model consisting of eight genes (SATB1, ZNF564, SOX14, EN1, IKZF2, SLC2A4RG, FOXJ2, and ZNF521). Patients in the high-risk group had a lower survival rate than those in the low-risk group. The area under the 3-year ROC curve of the model reached 0.825, suggesting a good predictive efficacy. We performed target gene prediction for the eight transcription factors in the model using six online databases and found that TUT1 may be a target gene for transcription factor EN1 and is associated with immune infiltration.

**Conclusion:**

This prognostic model consisting of eight transcription factor-associated genes demonstrated reliable predictive efficacy. This prediction model may provide new potential targets for the treatment of neuroblastoma and personalized monitoring of neuroblastoma patients with high and low risk.

## 1. Introduction

Neuroblastoma is the most common extracranial solid tumor of childhood, accounting for 8% of all pediatric tumors and also 15% of tumor-related deaths in children [1]. Neuroblastoma arises from neural crest precursor cells, which proliferate uncontrollably due to impaired differentiation [2]. The clinical diversity of neuroblastoma reflects its heterogeneous character. In some patients, the tumor regresses on its own, while others progress rapidly and become resistant to treatment. Although significant progress has been made in the treatment of neuroblastoma in recent years, recent studies have identified several molecules, such as MYCN, ALK, and ARID1B, involved in the development of neuroblastoma [3], the prognosis of neuroblastoma patients remains very poor, especially in cases of advanced or recurrent tumors. Therefore, there is an urgent need to discover novel markers with prognostic and predictive power in order to target and individualize the treatment of neuroblastoma patients.

Transcription factors (TFs) are a number of proteins that can specifically bind DNA sequences and regulate transcription [4]. TFs play an important role in tumorigenesis, progression, invasion, metastasis, and drug resistance [5]. Statistically, about 20% of oncogenes are TFs [6]. On the other hand, loss of function of TFs with tumorigenic suppressive effects leads to uncontrolled cell division and cancer development and progression [7]. However, there are few studies on TFs with prognostic values.

In this study, we constructed a prognostic model for patients with neuroblastoma based on TF-related genes by downloading transcriptomic data from the Therapeutically Applicable Research to Generate Effective Treatments (TARGET) database and validated it using the Gene Expression Omnibus (GEO) database. Our study found that our risk score could be an independent prognostic factor for neuroblastoma. In conclusion, our study shows that the prognostic model has a very high predictive value for neuroblastoma patients and provides a new potential target for the treatment of neuroblastoma patients.

## 2. Materials and Methods

### 2.1. Data Collection

**Figure 1:**
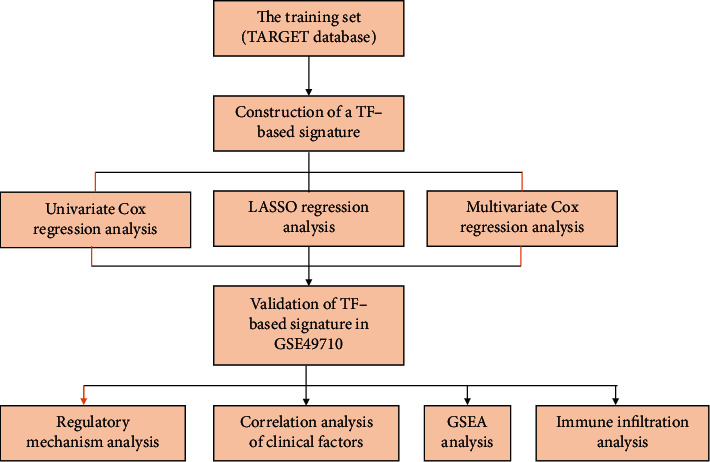
Flowchart of this study.

In 2018, Lambert et al. published a review that identified more than 1600 TFs that may be involved in human physiological and disease processes [8]. We collected 1639 TFs from the literature for analysis (Table S1).  Figure 1 shows the flow chart of this study. We downloaded transcriptomic data and clinical information from the TARGET database and the GEO database. Data from 148 patients in the TARGET database were used for model construction. Data from 498 neuroblastoma patients in the GEO dataset (GSE49710) were used for model validation.

### 2.2. Construction of the Prognostic TF-Based Signature

To screen TFs with prognostic value, we performed univariate Cox regression analysis of 1639 TFs to assess the correlation between TFs and overall survival (OS) in the TARGET dataset. We defined a screening criterion of Cox *P* < 0.01 for later analysis. To avoid overfitting between variables during model construction, we used the least absolute shrinkage and selection operator (LASSO) regression algorithm to extract the best subset. Finally, we obtained the coefficients of each incorporated TF in the model. Risk scores were calculated according to the following equation:

(1) $$\begin{array}{c} \text{risk score}=\sum_{i=1}^{N}\left(\text{Expi}*\text{Coei}\right). \end{array}$$

Expi represents the expression of each TF, and Coei is the regression coefficient of the corresponding multivariate Cox result.

To further validate the predictive performance of the TF-based prognostic model, we evaluated the AUC of the time-dependent ROC curves to assess the predictive value of risk scores on time-dependent outcomes.

### 2.3. Gene Set Enrichment Analysis (GSEA)

We used GSEA to analyze the potentially different biological mechanisms between the high- and low-risk groups. The GSEA was annotated using the “clusterProfiler” package in R software.

### 2.4. Immune Infiltration Analysis

We calculated the level of 22 tumor-infiltrating immune cells by CIBERSORT algorithm [9], a method to calculate a certain cell composition from the gene expression profile of a tissue. In the present study, we used the CIBERSORT algorithm to calculate the level of immune cell infiltration in tumor samples from the TARGET and GEO databases.

### 2.5. Statistical Analysis

We used R software (version 3.8.2) to complete all statistical analyses. Student's *t*-test was employed to examine statistically significant differences between groups, while one-way ANOVA was used for the comparison of differences between groups. The chi-square test was employed to compare the clinical features of the two groups. Neuroblastoma patients were analyzed using the Kaplan–Meier technique and the log-rank test to determine their overall survival time (OS). The predictive value of risk score and clinical characteristics was assessed by using one-way and Cox regression analysis. Spearman analysis was used to analyze the correlation between the two variables (*P* < 0.05).

## 3. Results

### 3.1. Identifying the Potential Prognostic Transcription Factor

We obtained a list of 1639 human TFs from the public literature [8, 10]. After corresponding to the TARGET database, a total of 1375 TFs had gene expression data for inclusion in the follow-up study. Information on the clinical characteristics of neuroblastoma patients in both datasets is in Table S2.

Univariate Cox regression analysis of the gene expression was performed, and we searched for TFs with prognostic significance from 1375 TFs. 65 TFs with *P* ≤ 0.01 were obtained for the next analysis (Table S3).

### 3.2. Constructing the TF-Based Predictive Model

**Figure 2:**
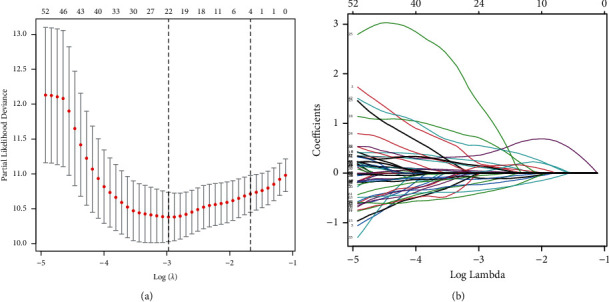
65 TFs with significant prognostic value subjected to LASSO regression analysis. (a) Each line represents a TF with a significant prognostic value. (b) Plot of partial likelihood deviation.

To avoid overfitting between variables during model construction, we used the LASSO regression algorithm to extract the best subset (Figure 2). Then, we obtained the coefficients of each TF in the prognostic model by multivariate Cox regression analysis (Table 1), consisting of 8 genes that constituted this prognostic model. The coefficients of ZNF564, SOX14, EN1, and SLC2A4RG were positive and were risk genes for poor prognosis. In contrast, the coefficients of SATB1, IKZF2, FOXJ2, and ZNF521 were negative and were protective factors.

**Figure 3:**
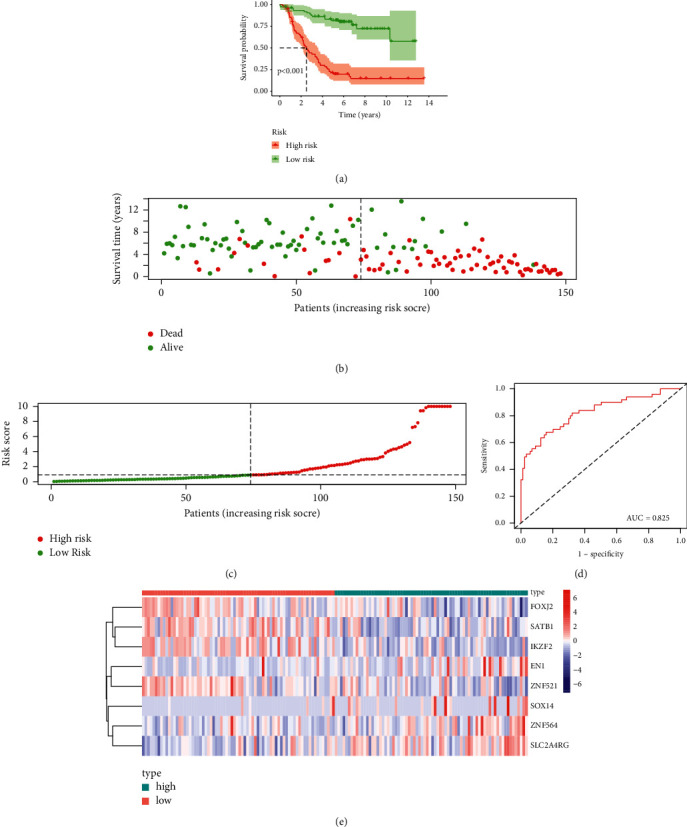
Characteristics of the prognostic model consisting of eight TFs in the TARGET dataset. (a) Kaplan–Meier survival analysis of the TF-based prognostic models. (b) Distribution of survival times of neuroblastoma patients. (c) Distribution of risk scores of neuroblastoma patients. (d) The AUC value reflects the high prognostic accuracy of the TF-based signature. (e) Distribution of the expression values of TFs in the model.

We divided the patients into high- and low-risk groups by median risk score (Figure 3(c)). High-risk patients had a higher mortality rate than low-risk patients (Figure 3(b)). From the heat map, we found that ZNF564, SOX14, EN1, and SLC2A4RG had increased expression in high-risk group, while SATB1, IKZF2, FOXJ2, and ZNF521 had increased expression in the low-risk group (Figure 3(e)).  Figure 3(a) shows the Kaplan–Meier curves for the two groups, with longer OS in low-risk patients than the other groups. Our sensitivity and specificity evaluation of the model was performed by time-dependent ROC analysis. In the 3-year ROC curve, the AUC was 0.825, indicating the good predictive performance of the prediction model for 3-year OS (Figure 3(d)).

### 3.3. Validation of the TF-Based Signature

**Figure 4:**
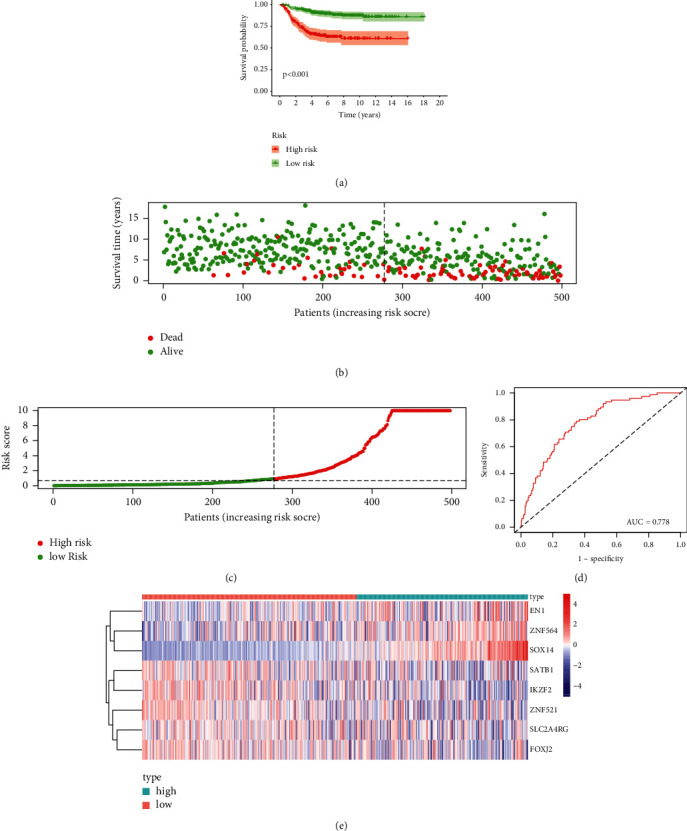
Validation of the eight TF-based risk signatures in GEO cohort. (a) Kaplan–Meier survival analysis of the TF-based prognostic models. (b) Distribution of survival times of neuroblastoma patients. (c) Distribution of risk scores of neuroblastoma patients. (d) Area under the curve reflects the high prognostic accuracy of the TF-based signature. (e) Distribution of the expression values of TFs in the model.

The prediction model we constructed was validated in another dataset GSE49710. We used the same prognostic model obtained from the TARGET dataset to calculate risk scores for a total of 498 patients in the GSE49710 dataset. The same division into low- and high-risk groups was done according to the median risk score. In GSE49710, the sensitivity and specificity of our TF-based prognostic model for 3-year OS were evaluated as 0.778 (Figure 4(c)). Other findings, including heat map and Kaplan–Meier analysis, were also consistent with the results of the TARGET cohort, suggesting that our TF-based prognostic model has good stability in neuroblastoma patients (Figures 4(a), 4(b), 4(d), and 4(e)).

### 3.4. The Identification of the TF-Based Signature's Independent Predictive Activity

**Figure 5:**
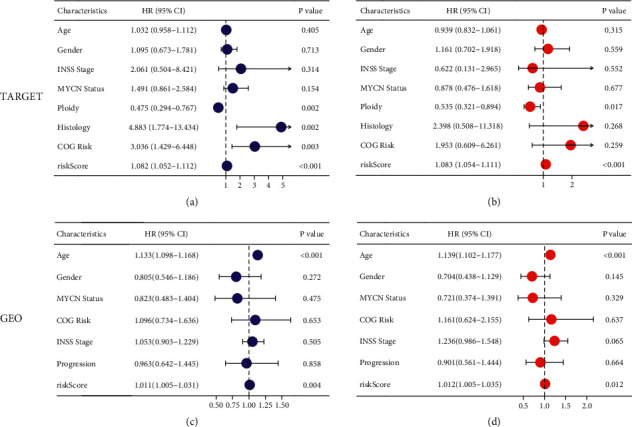
Univariate (a, c) and multivariate (b, d) Cox regression analyses in OS of neuroblastoma patients among the gene signature and clinicopathological factors. (a, b) TARGET cohort. (c, d) GEO cohort.

To further investigate whether the TF-based prognostic model can independently predict the prognosis of neuroblastoma patients. Univariate Cox regression analysis of whether the TF-based prognostic model could independently predict other clinicopathological characteristics showed that ploidy, histology, COG risk, and risk score were associated with OS in the TARGET dataset (Figure 5(a)). We then included the variables in a multivariate Cox regression analysis showing that ploidy and risk score remained independent predictors of OS (Figure 5(b)). In GSE49710, age and risk score were associated with OS (Figure 5(c)), and age and risk score remained independent prognostic indicators of OS after multivariate Cox regression (Figure 5(d)).

### 3.5. GSEA Identifies Biological Pathways

**Figure 6:**
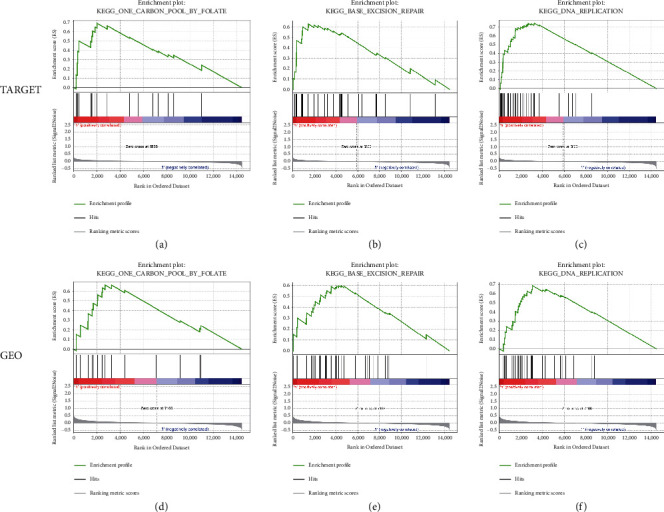
GSEA analysis of the high-risk group in both TARGET cohort (a–c) and GEO cohort (d–f).

To further investigate the relevant signaling pathways in the high-risk group, we performed GSEA analysis between the high-risk and low-risk groups. In the high-risk group of the TARGET database, one carbon pool by folate, base excision repair, and DNA replication were enriched (Figures 6(a)–6(c)). These were further validated in the GEO database (Figures 6(d)–6(f)).

### 3.6. The Regulatory Mechanism Analysis Based on Six Databases

To validate this transcriptional regulatory relationship, we used six online databases, including JASPAR, ENCODE, CHEA, MotifMap, TRANSFAC, and TRRUST. We made predictions for the target genes of the eight TFs in the model (Table S4), listing evidence supported by more than two databases (Table 2). “√” indicated that the corresponding TF has a regulatory relationship with the target gene.

### 3.7. Survival Analysis of TUT1 and Correlation with Immune Infiltration

**Figure 7:**
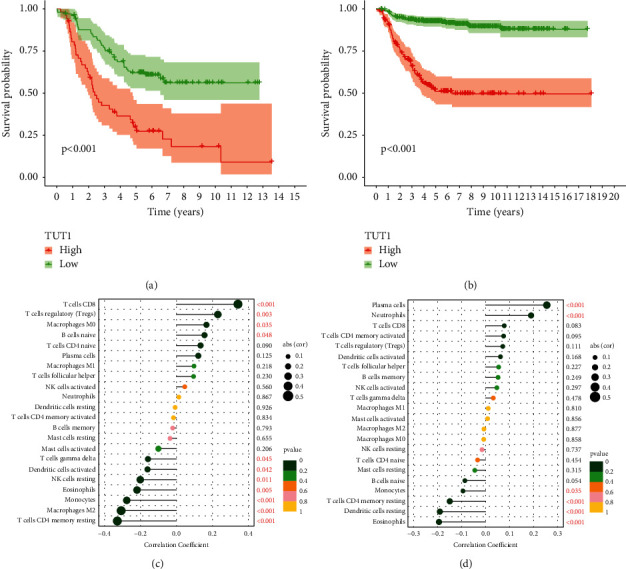
Survival analysis of TUT1 and correlation between TUT1 and infiltration immune cells. (a, c) TARGET cohort. (b, d) GEO cohort.

Interestingly, we found that the higher the expression of TUT1, the shorter the OS of the patients in both TARGET and GEO datasets (Figures 7(a) and 7(b)). By using CIBERSORT to calculate immune cell infiltrations, we found that TUT1 was positively correlated with T cells CD8, T cells regulatory (Tregs), macrophages M0, B cells naive and negatively correlated with T cells CD4 memory resting, macrophages M2, monocytes, eosinophils, NK cells resting, dendritic cells activated, and T cells gamma delta in TARGET database (Figure 7(c)). We also found that TUT1 was positively correlated with plasma cells and neutrophils and negatively correlated with eosinophils, dendritic cells resting, T cells CD4 memory resting, and monocytes in GEO database (Figure 7(d)). There may be a role for TUT1 in the development of neuroblastoma, and the transcription factor EN1 may also be regulated in relation to the predicted target gene TUT1. This needs to be further elucidated.

## 4. Discussion

In this study, we showed that the TFs-based prognostic model we constructed could predict neuroblastoma patients well. TFs have a role in gene transcription as well as a variety of other critical biological activities [11, 12]. A third of all human developmental diseases are linked to aberrant TF expression [13]. Dong et al. discovered that transcription factors might enhance breast cancer development [14]. A crucial involvement for transcription factors in hepatoblastomas was found by Zhan and colleagues [15]. TFs have also been implicated with neuroblastoma in several investigations [16]. However, there are few studies on the prognostic aspects of transcription factors. As effector molecules of cell signaling pathways, TFs play an important role in tumor development, and therefore, it is necessary to study their functions in predicting patient prognosis. We analyzed data from TARGET and GEO databases by bioinformatics methods to construct and validate the role of prognostic models and analyze their association with clinical features and immune infiltration and also predict target genes of transcription factors. We used the data from TARGET for model construction and the dataset from GEO for model validation based on 8 TFs using Cox regression and LASSO regression methods. This signature can well predict the prognosis of patients with neuroblastoma.

In this study, a prognostic model consisting of eight TFs whose roles in tumors have been partially reported was constructed. SATB1 is capable of regulating chromatin structure and gene expression through chromatin remodeling enzymes. SATB1 is expressed in a diverse population of adult progenitor cells and embryonic stem cells. This gene has been linked to a number of various forms of cancers, such as laryngeal squamous-cell carcinoma [17], endometrial cancer [18], hepatocellular carcinoma [19, 20], rectal cancer [21], melanoma [22], and gastric cancer [23, 24]. SOX14 is a member of the SOXB2 transcription factor subgroup, and Li et al. reported that through the Wnt/-catenin pathway [25], SOXQ4 might enhance cervical cancer cell proliferation and invasion. EN1 plays a role in abnormal expression of EN1 is common in colorectal cancer [26], prostate cancer [27], and astrocytoma [28]. IKZF2 is a member of the Ikaros family of transcription factors and has been shown to be a transcription factor essential for regulatory T-cell function [29, 30]. Park et al. reported that IKZF2 could inhibit myeloid differentiation by driving the self-renewal of leukemic stem cells [31]. It has been shown by Zhao et al. that SLC2A4RG encodes a nuclear transcription factor that helps activate the solute carrier family two member four gene, a gene that may have a role in the formation of GBM [32]. In addition to being a member of the FOX family, FOXJ2 is a new forkhead factor with dual DNA binding specificity. Breast cancer cell migration and invasion may be inhibited if FOXJ2 expression is increased [33]. ZNF521 is a transcription factor involved in the regulation of hematopoietic, neural, and mesenchymal stem cells, and Chiarella et al. reported that ZNF521 inhibits the differentiation of human adipose-derived stem cells [34]. These studies suggest that these transcription factors are closely associated with tumors. Our prognostic model can well predict the prognosis of neuroblastoma patients.

We also used six databases to predict our eight TF target genes and found that the TUT1 gene not only has prognostic value but was found to be associated with immune cells by the CIBERSORT algorithm, which was validated in both the TARGET database and the GEO database. This gene encodes a nucleotidyltransferase that functions as both a terminal uridylyltransferase and a nuclear poly(A) polymerase. The encoded enzyme specifically adds and removes nucleotides from the 3′ end of small nuclear RNAs and select mRNAs and may function in controlling gene expression and cell proliferation. This provides a direction for further research in the future. Although our model was able to predict the prognosis of neuroblastoma patients well, our study still has some shortcomings. Our prognostic model still needs clinical cases for further validation, not just using data on public databases. Some of the TFs in our model has not been studied in neuroblastoma with relevant mechanisms, which need to be refined in our future studies.

## 5. Conclusion

Our study successfully constructed a prognostic model containing eight TFs (ZNF564, SOX14, EN1, SLC2A4RG, SATB1, IKZF2, FOXJ2, and ZNF521). Our prognostic model can help clinicians predict OS in neuroblastoma patients, but further studies with clinical samples are needed to validate the accuracy of our prognostic model to improve the management of neuroblastoma.

## Figures and Tables

**Table 1:** Eight TF-related genes identified by multivariate Cox regression analysis.

Id	coef	HR	HR.95L	HR.95H	*P* value
SATB1	−0.56897	0.566111	0.365756	0.876216	0.010684
ZNF564	1.370636	3.937853	2.136288	7.258708	1.12E-05
SOX14	3.153827	23.42554	2.019831	271.6841	0.011663
EN1	0.34383	1.410339	1.180563	1.684837	0.000151
IKZF2	−0.87769	0.415742	0.239868	0.720567	0.001761
SLC2A4RG	1.118871	3.061396	1.680095	5.578342	0.000257
FOXJ2	−0.65706	0.518375	0.313321	0.857627	0.010532
ZNF521	−0.66938	0.512027	0.383883	0.682947	5.24E-06

**Table 2:** Predictions for the target genes of the eight transcription factors included in the model supported by more than two public databases.

TF	Gene	CHEA	ENCODE	JASPAR	MotifMap	TRANSFAC	TRRUST
EN1	TUT1	—	—	√	—	√	—
EN1	SAV1	—	—	√	—	√	—
EN1	PAX6	—	—	√	—	√	—
EN1	ATP13A4	—	—	√	—	√	—

## Data Availability

All of the data used in this study are available online.

## Supplementary Materials

Supplementary Materials Table S1: 1639 TFs from public literature. Table S2: clinical and pathologic factors of the datasets used in this study. Table S3: 65 TFs with *P* ≤ 0.01 after univariate Cox regression. Table S4: predictions for the target genes of the eight TFs.
