# Supplementary material for: Identification and External Validation of a Transcription Factor-Related Prognostic Signature in Pediatric Neuroblastoma
Source: J Oncol. 2021 Dec 28;2021:1370451. doi: 10.1155/2021/1370451 (PMC8727167; doi:10.1155/2021/1370451)
Supplement: Supplementary Materials — Table S1: 1639 TFs from public literature. Table S2: clinical and pathologic factors of the datasets used in this study. Table S3: 65 TFs with P ≤ 0.01 after univariate Cox regression. Table S4: predictions for the target genes of the eight TFs. [file 1370451.f1.zip › 1370451.f1/Table S2 (1).docx]

Table S2. Clinical and pathologic factors of the datasets used in this study.

| Characteristics |  | TARGET(%) | GSE49710(%) |
| --- | --- | --- | --- |
| Gender | Male | 60(40.5%) | 287(57.7%) |
|  | Female | 88(59.5%) | 211(42.3) |
| Age | <18 months | 27(18.2%) | 300(60.2%) |
|  | ≥18 months | 121(81.8%) | 198(39.8%) |
| INSS Stage | Stage 1 | 0(0%) | 121(24.3%) |
|  | Stage 2 | 1(0.7% | 78(15.7%) |
|  | Stage 3 | 6(4.1%) | 63(12.7%) |
|  | Stage 4 | 121(81.7%) | 183(36.7%) |
|  | Stage 4S | 20(13.5%) | 53(10.6%) |
| MYCN status | No amplification | 114(77.1%) | 401(80.5%) |
|  | Amplification | 33(22.2%) | 92(18.5%) |
|  | Unknown | 1(0.7%) | 5(10.0%) |
| Ploidy | 1 | 62(41.9%) | / |
|  | >1 | 85(57.4%) | / |
|  | Unknown | 1(0.7%) | / |
| Histology | Favorable | 26(17.6%) | / |
|  | Unfavorable | 111(75%) | / |
|  | Unknown | 11(7.4%) | / |
| Grade | Differentiating | 9(6.1%) | / |
|  | Undifferentiating | 116(78.4%) | / |
|  | Unknown | 23(15.5%) | / |
| MKI | Low | 45(30.4%) | / |
|  | Intermediate | 40(27.0%) | / |
|  | High | 33(22.3%) | / |
|  | Unknown | 30(20.3%) | / |
| COG risk | High risk | 122(82.4%) | / |
|  | Intermediate risk | 13(8.8%) | / |
|  | Low risk | 13(8.8%) | / |
| Class label | Favorable | / | 181(36.3%) |
|  | Unfavorable | / | 91(18.3%) |
|  | Unknown | / | 226(45.4%) |
| Progression | No | / | 315(63.3%) |
|  | Yes | / | 183(36.7) |
| High risk | No | / | 322(64.6%) |
|  | Yes | / | 176(35.4%) |
| Total |  | 148(100%) | 498(100%) |
