# Supplementary material for: Identification and External Validation of a Transcription Factor-Related Prognostic Signature in Pediatric Neuroblastoma
Source: J Oncol. 2021 Dec 28;2021:1370451. doi: 10.1155/2021/1370451 (PMC8727167; doi:10.1155/2021/1370451)
Supplement: Supplementary Materials — Table S1: 1639 TFs from public literature. Table S2: clinical and pathologic factors of the datasets used in this study. Table S3: 65 TFs with P ≤ 0.01 after univariate Cox regression. Table S4: predictions for the target genes of the eight TFs. [file 1370451.f1.zip › 1370451.f1/Table S3 (1).docx]

Table S3. 65 TFs with p≤0.01 after univariate Cox regression.

| id | HR | HR.95L | HR.95H | pvalue |
| --- | --- | --- | --- | --- |
| ZNF624 | 0.556939 | 0.38361 | 0.808583 | 0.002091 |
| MAZ | 1.827315 | 1.204037 | 2.773238 | 0.004621 |
| CHCHD3 | 2.100035 | 1.296162 | 3.402466 | 0.002582 |
| HIVEP2 | 0.618213 | 0.442574 | 0.863554 | 0.004799 |
| DNTTIP1 | 2.644744 | 1.467406 | 4.766692 | 0.001213 |
| E2F4 | 2.429874 | 1.293911 | 4.563135 | 0.005756 |
| ZNF628 | 1.957831 | 1.290096 | 2.971175 | 0.001595 |
| ZNF101 | 1.675318 | 1.156046 | 2.427838 | 0.00641 |
| DRAP1 | 2.058783 | 1.394096 | 3.040383 | 0.000283 |
| SOX12 | 1.857859 | 1.183251 | 2.91708 | 0.007125 |
| SATB1 | 0.56842 | 0.393369 | 0.821371 | 0.002632 |
| ZNF598 | 4.075527 | 2.115042 | 7.853233 | 2.69E-05 |
| MESP1 | 2.196377 | 1.470839 | 3.279809 | 0.00012 |
| NKX6-2 | 1.220673 | 1.096029 | 1.359493 | 0.000285 |
| ATF2 | 0.462291 | 0.292104 | 0.731635 | 0.000988 |
| MYBL2 | 1.319565 | 1.077411 | 1.616144 | 0.007345 |
| ETV5 | 1.32576 | 1.090944 | 1.61112 | 0.004581 |
| ZNF564 | 2.320682 | 1.395191 | 3.860091 | 0.001184 |
| DBX2 | 0.377946 | 0.204794 | 0.697497 | 0.001856 |
| CENPB | 4.394624 | 2.309691 | 8.361602 | 6.47E-06 |
| MBNL2 | 0.592405 | 0.425687 | 0.824417 | 0.001903 |
| ZNF787 | 4.160209 | 2.602054 | 6.651414 | 2.61E-09 |
| ZBTB38 | 0.568536 | 0.389218 | 0.830469 | 0.003491 |
| ZNF764 | 2.259069 | 1.301689 | 3.920594 | 0.003763 |
| SOX14 | 18.01433 | 2.063884 | 157.2356 | 0.008911 |
| TSHZ2 | 0.685763 | 0.52861 | 0.889638 | 0.004504 |
| EN1 | 1.213279 | 1.056987 | 1.392681 | 0.006002 |
| ZBTB4 | 0.594161 | 0.401215 | 0.879895 | 0.009359 |
| IKZF2 | 0.410103 | 0.254845 | 0.659948 | 0.000241 |
| BAZ2B | 0.500616 | 0.331975 | 0.754928 | 0.000962 |
| ZNF768 | 2.246157 | 1.396815 | 3.611948 | 0.000841 |
| E2F1 | 1.305981 | 1.072459 | 1.590352 | 0.007909 |
| WIZ | 2.346865 | 1.32325 | 4.162308 | 0.003522 |
| GATAD2A | 1.949506 | 1.259911 | 3.016541 | 0.002724 |
| ZNF562 | 2.08887 | 1.269482 | 3.437131 | 0.003743 |
| PRDM2 | 0.509247 | 0.339888 | 0.762994 | 0.001071 |
| HIF3A | 1.619361 | 1.165675 | 2.249624 | 0.004053 |
| HMGA1 | 1.877608 | 1.2544 | 2.810439 | 0.002203 |
| ZNF358 | 2.038989 | 1.314018 | 3.163943 | 0.001482 |
| IKZF5 | 0.469552 | 0.302913 | 0.727862 | 0.000724 |
| FIZ1 | 2.464986 | 1.42679 | 4.25862 | 0.00122 |
| SLC2A4RG | 3.163834 | 1.83765 | 5.44709 | 3.25E-05 |
| ELK1 | 1.550596 | 1.166964 | 2.060345 | 0.002489 |
| ZNF25 | 0.619077 | 0.445614 | 0.860064 | 0.004255 |
| MBD3 | 2.106142 | 1.284539 | 3.45325 | 0.003152 |
| ZNF121 | 1.467197 | 1.126845 | 1.910349 | 0.004416 |
| KLF11 | 0.609291 | 0.423046 | 0.877528 | 0.007771 |
| ZNF804A | 0.805149 | 0.695033 | 0.932712 | 0.003873 |
| ZBTB3 | 2.949144 | 1.829939 | 4.752861 | 8.92E-06 |
| KLF12 | 0.652954 | 0.476733 | 0.894313 | 0.007908 |
| MXD3 | 1.701835 | 1.172739 | 2.469641 | 0.005131 |
| THAP11 | 1.983891 | 1.181283 | 3.33182 | 0.009604 |
| FOXJ2 | 0.378922 | 0.231238 | 0.620928 | 0.000118 |
| ZNF557 | 2.202786 | 1.366582 | 3.550661 | 0.001186 |
| ZNF511 | 2.939346 | 1.773057 | 4.8728 | 2.91E-05 |
| ZNF219 | 1.728222 | 1.188646 | 2.512733 | 0.004171 |
| ZNF436 | 0.498524 | 0.344848 | 0.720681 | 0.000214 |
| HES6 | 1.743672 | 1.261707 | 2.409747 | 0.000756 |
| ZNF668 | 2.179183 | 1.235191 | 3.844621 | 0.007163 |
| NME2 | 2.552591 | 1.726565 | 3.773804 | 2.63E-06 |
| L3MBTL4 | 0.71858 | 0.565089 | 0.913762 | 0.007027 |
| NR2F6 | 2.333597 | 1.55354 | 3.505331 | 4.46E-05 |
| ZNF521 | 0.595281 | 0.454712 | 0.779305 | 0.00016 |
| ZNF771 | 2.255365 | 1.482975 | 3.430045 | 0.000143 |
| PBX1 | 0.585041 | 0.393301 | 0.870257 | 0.008149 |
