# Supplementary material for: Identification and External Validation of a Transcription Factor-Related Prognostic Signature in Pediatric Neuroblastoma
Source: J Oncol. 2021 Dec 28;2021:1370451. doi: 10.1155/2021/1370451 (PMC8727167; doi:10.1155/2021/1370451)
Supplement: Supplementary Materials — Table S1: 1639 TFs from public literature. Table S2: clinical and pathologic factors of the datasets used in this study. Table S3: 65 TFs with P ≤ 0.01 after univariate Cox regression. Table S4: predictions for the target genes of the eight TFs. [file 1370451.f1.zip › 1370451.f1/Table S4.docx]

Table S4. Predictions for the target genes of the eight TFs.

| TF | Gene | CHEA | ENCODE | JASPAR | MotifMap | TRANSFAC | TRRUST | Sum |
| --- | --- | --- | --- | --- | --- | --- | --- | --- |
| EN1 | TUT1 | 0 | 0 | 1 | 0 | 1 | 0 | 2 |
| EN1 | SAV1 | 0 | 0 | 1 | 0 | 1 | 0 | 2 |
| EN1 | PAX6 | 0 | 0 | 1 | 0 | 1 | 0 | 2 |
| EN1 | ATP13A4 | 0 | 0 | 1 | 0 | 1 | 0 | 2 |
| EN1 | NCAM1 | 0 | 0 | 1 | 0 | 0 | 0 | 1 |
| FOXJ2 | SMARCD1 | 0 | 0 | 0 | 0 | 1 | 0 | 1 |
| EN1 | PSAP | 0 | 0 | 1 | 0 | 0 | 0 | 1 |
| FOXJ2 | NEIL3 | 0 | 0 | 0 | 0 | 1 | 0 | 1 |
| FOXJ2 | UBR1 | 0 | 0 | 0 | 0 | 1 | 0 | 1 |
| EN1 | TRMT44 | 0 | 0 | 1 | 0 | 0 | 0 | 1 |
| EN1 | USP13 | 0 | 0 | 1 | 0 | 0 | 0 | 1 |
| FOXJ2 | HSF2 | 0 | 0 | 0 | 0 | 1 | 0 | 1 |
| EN1 | P2RX4 | 0 | 0 | 1 | 0 | 0 | 0 | 1 |
| FOXJ2 | SOBP | 0 | 0 | 0 | 0 | 1 | 0 | 1 |
| EN1 | EIF4EBP2 | 0 | 0 | 1 | 0 | 0 | 0 | 1 |
| EN1 | MAGEA2 | 0 | 0 | 1 | 0 | 0 | 0 | 1 |
| FOXJ2 | MNAT1 | 0 | 0 | 0 | 0 | 1 | 0 | 1 |
| EN1 | ZFAT | 0 | 0 | 1 | 0 | 0 | 0 | 1 |
| EN1 | ADRBK2 | 0 | 0 | 1 | 0 | 0 | 0 | 1 |
| EN1 | UBAP1L | 0 | 0 | 1 | 0 | 0 | 0 | 1 |
| FOXJ2 | BAI3 | 0 | 0 | 0 | 0 | 1 | 0 | 1 |
| EN1 | GCNT3 | 0 | 0 | 1 | 0 | 0 | 0 | 1 |
| FOXJ2 | POU4F2 | 0 | 0 | 0 | 0 | 1 | 0 | 1 |
| EN1 | WDR48 | 0 | 0 | 1 | 0 | 0 | 0 | 1 |
| FOXJ2 | BLID | 0 | 0 | 0 | 0 | 1 | 0 | 1 |
| FOXJ2 | LGI1 | 0 | 0 | 0 | 0 | 1 | 0 | 1 |
| EN1 | CEP97 | 0 | 0 | 1 | 0 | 0 | 0 | 1 |
| EN1 | LCOR | 0 | 0 | 1 | 0 | 0 | 0 | 1 |
| EN1 | PTGER2 | 0 | 0 | 1 | 0 | 0 | 0 | 1 |
| EN1 | HMBS | 0 | 0 | 1 | 0 | 0 | 0 | 1 |
| EN1 | BEND7 | 0 | 0 | 1 | 0 | 0 | 0 | 1 |
| EN1 | KRTAP4-4 | 0 | 0 | 1 | 0 | 0 | 0 | 1 |
| EN1 | TOMM34 | 0 | 0 | 1 | 0 | 0 | 0 | 1 |
| EN1 | PLBD2 | 0 | 0 | 1 | 0 | 0 | 0 | 1 |
| EN1 | TSHZ3 | 0 | 0 | 0 | 0 | 1 | 0 | 1 |
| EN1 | GZMH | 0 | 0 | 1 | 0 | 0 | 0 | 1 |
| EN1 | SAT1 | 0 | 0 | 0 | 0 | 1 | 0 | 1 |
| FOXJ2 | TNF | 0 | 0 | 0 | 0 | 1 | 0 | 1 |
| FOXJ2 | NCAM1 | 0 | 0 | 0 | 0 | 1 | 0 | 1 |
| EN1 | NXF1 | 0 | 0 | 0 | 0 | 1 | 0 | 1 |
| EN1 | BNIP1 | 0 | 0 | 1 | 0 | 0 | 0 | 1 |
| EN1 | ACSM2A | 0 | 0 | 1 | 0 | 0 | 0 | 1 |
| EN1 | MDFI | 0 | 0 | 1 | 0 | 0 | 0 | 1 |
| EN1 | ZBTB21 | 0 | 0 | 1 | 0 | 0 | 0 | 1 |
| FOXJ2 | SSH3 | 0 | 0 | 0 | 0 | 1 | 0 | 1 |
| FOXJ2 | IMPDH1 | 0 | 0 | 0 | 0 | 1 | 0 | 1 |
| EN1 | RPL38 | 0 | 0 | 0 | 0 | 1 | 0 | 1 |
| FOXJ2 | KCNK10 | 0 | 0 | 0 | 0 | 1 | 0 | 1 |
| EN1 | PLEKHB2 | 0 | 0 | 1 | 0 | 0 | 0 | 1 |
| EN1 | RAB11FIP3 | 0 | 0 | 1 | 0 | 0 | 0 | 1 |
| FOXJ2 | SDHC | 0 | 0 | 0 | 0 | 1 | 0 | 1 |
| EN1 | SMYD1 | 0 | 0 | 1 | 0 | 0 | 0 | 1 |
| FOXJ2 | TYRO3 | 0 | 0 | 0 | 0 | 1 | 0 | 1 |
| EN1 | KLK5 | 0 | 0 | 1 | 0 | 0 | 0 | 1 |
| FOXJ2 | PPARGC1A | 0 | 0 | 0 | 0 | 1 | 0 | 1 |
| EN1 | GOLGA7 | 0 | 0 | 1 | 0 | 0 | 0 | 1 |
| FOXJ2 | SCUBE3 | 0 | 0 | 0 | 0 | 1 | 0 | 1 |
| EN1 | ABHD13 | 0 | 0 | 1 | 0 | 0 | 0 | 1 |
| FOXJ2 | ZBTB37 | 0 | 0 | 0 | 0 | 1 | 0 | 1 |
| FOXJ2 | PIK3C2A | 0 | 0 | 0 | 0 | 1 | 0 | 1 |
| EN1 | ADAMTSL3 | 0 | 0 | 1 | 0 | 0 | 0 | 1 |
| EN1 | NEDD4L | 0 | 0 | 1 | 0 | 0 | 0 | 1 |
| EN1 | LRP3 | 0 | 0 | 1 | 0 | 0 | 0 | 1 |
| FOXJ2 | ARPP21 | 0 | 0 | 0 | 0 | 1 | 0 | 1 |
| SATB1 | IL4 | 0 | 0 | 0 | 0 | 0 | 1 | 1 |
| EN1 | NEK1 | 0 | 0 | 1 | 0 | 0 | 0 | 1 |
| EN1 | GAS7 | 0 | 0 | 1 | 0 | 0 | 0 | 1 |
| EN1 | MCHR1 | 0 | 0 | 1 | 0 | 0 | 0 | 1 |
| FOXJ2 | R3HDM2 | 0 | 0 | 0 | 0 | 1 | 0 | 1 |
| EN1 | IFRD1 | 0 | 0 | 1 | 0 | 0 | 0 | 1 |
| EN1 | KLRG2 | 0 | 0 | 1 | 0 | 0 | 0 | 1 |
| FOXJ2 | USP2 | 0 | 0 | 0 | 0 | 1 | 0 | 1 |
| EN1 | PRSS53 | 0 | 0 | 1 | 0 | 0 | 0 | 1 |
| EN1 | CWC27 | 0 | 0 | 1 | 0 | 0 | 0 | 1 |
| EN1 | KLF1 | 0 | 0 | 1 | 0 | 0 | 0 | 1 |
| FOXJ2 | CDH20 | 0 | 0 | 0 | 0 | 1 | 0 | 1 |
| EN1 | LIPT2 | 0 | 0 | 1 | 0 | 0 | 0 | 1 |
| EN1 | SP2 | 0 | 0 | 1 | 0 | 0 | 0 | 1 |
| FOXJ2 | HOXC4 | 0 | 0 | 0 | 0 | 1 | 0 | 1 |
| EN1 | NLGN1 | 0 | 0 | 1 | 0 | 0 | 0 | 1 |
| FOXJ2 | GNAO1 | 0 | 0 | 0 | 0 | 1 | 0 | 1 |
| EN1 | GFI1B | 0 | 0 | 1 | 0 | 0 | 0 | 1 |
| EN1 | OR2H2 | 0 | 0 | 1 | 0 | 0 | 0 | 1 |
| EN1 | GGT5 | 0 | 0 | 1 | 0 | 0 | 0 | 1 |
| EN1 | PCOLCE2 | 0 | 0 | 1 | 0 | 0 | 0 | 1 |
| EN1 | PSMG3 | 0 | 0 | 1 | 0 | 0 | 0 | 1 |
| EN1 | LMAN2 | 0 | 0 | 1 | 0 | 0 | 0 | 1 |
| EN1 | EYA4 | 0 | 0 | 0 | 0 | 1 | 0 | 1 |
| FOXJ2 | CXCL2 | 0 | 0 | 0 | 0 | 1 | 0 | 1 |
| FOXJ2 | CDH10 | 0 | 0 | 0 | 0 | 1 | 0 | 1 |
| EN1 | CEND1 | 0 | 0 | 1 | 0 | 0 | 0 | 1 |
| FOXJ2 | FLOT1 | 0 | 0 | 0 | 0 | 1 | 0 | 1 |
| EN1 | CDK19 | 0 | 0 | 1 | 0 | 0 | 0 | 1 |
| FOXJ2 | DMC1 | 0 | 0 | 0 | 0 | 1 | 0 | 1 |
| EN1 | FARS2 | 0 | 0 | 1 | 0 | 0 | 0 | 1 |
| EN1 | TLR8 | 0 | 0 | 1 | 0 | 0 | 0 | 1 |
| EN1 | CD33 | 0 | 0 | 1 | 0 | 0 | 0 | 1 |
| FOXJ2 | FEZ1 | 0 | 0 | 0 | 0 | 1 | 0 | 1 |
| EN1 | SNORD65 | 0 | 0 | 1 | 0 | 0 | 0 | 1 |
| FOXJ2 | CEND1 | 0 | 0 | 0 | 0 | 1 | 0 | 1 |
| FOXJ2 | DIXDC1 | 0 | 0 | 0 | 0 | 1 | 0 | 1 |
| FOXJ2 | LUC7L3 | 0 | 0 | 0 | 0 | 1 | 0 | 1 |
| EN1 | C11orf31 | 0 | 0 | 1 | 0 | 0 | 0 | 1 |
| FOXJ2 | LTBP1 | 0 | 0 | 0 | 0 | 1 | 0 | 1 |
| FOXJ2 | FAM105A | 0 | 0 | 0 | 0 | 1 | 0 | 1 |
| EN1 | OTP | 0 | 0 | 0 | 0 | 1 | 0 | 1 |
| EN1 | OR11H4 | 0 | 0 | 1 | 0 | 0 | 0 | 1 |
| EN1 | GRHL2 | 0 | 0 | 0 | 0 | 1 | 0 | 1 |
| FOXJ2 | RBFOX1 | 0 | 0 | 0 | 0 | 1 | 0 | 1 |
| EN1 | LIPM | 0 | 0 | 1 | 0 | 0 | 0 | 1 |
| EN1 | TTC12 | 0 | 0 | 0 | 0 | 1 | 0 | 1 |
| EN1 | THBD | 0 | 0 | 1 | 0 | 0 | 0 | 1 |
| EN1 | KDELC1 | 0 | 0 | 1 | 0 | 0 | 0 | 1 |
| EN1 | PLA2G2A | 0 | 0 | 1 | 0 | 0 | 0 | 1 |
| FOXJ2 | C1ORF87 | 0 | 0 | 0 | 0 | 1 | 0 | 1 |
| EN1 | WDTC1 | 0 | 0 | 1 | 0 | 0 | 0 | 1 |
| EN1 | CDX2 | 0 | 0 | 0 | 0 | 1 | 0 | 1 |
| EN1 | TRIM7 | 0 | 0 | 1 | 0 | 0 | 0 | 1 |
| EN1 | FOXP2 | 0 | 0 | 0 | 0 | 1 | 0 | 1 |
| EN1 | SLC6A9 | 0 | 0 | 0 | 0 | 1 | 0 | 1 |
| EN1 | EMILIN2 | 0 | 0 | 1 | 0 | 0 | 0 | 1 |
| EN1 | KDM4C | 0 | 0 | 1 | 0 | 0 | 0 | 1 |
| EN1 | BCOR | 0 | 0 | 0 | 0 | 1 | 0 | 1 |
| FOXJ2 | FEN1 | 0 | 0 | 0 | 0 | 1 | 0 | 1 |
| EN1 | NPR3 | 0 | 0 | 0 | 0 | 1 | 0 | 1 |
| EN1 | LDLR | 0 | 0 | 1 | 0 | 0 | 0 | 1 |
| EN1 | NIM1K | 0 | 0 | 1 | 0 | 0 | 0 | 1 |
| EN1 | CERKL | 0 | 0 | 1 | 0 | 0 | 0 | 1 |
| EN1 | RAB3IL1 | 0 | 0 | 1 | 0 | 0 | 0 | 1 |
| EN1 | F2RL2 | 0 | 0 | 1 | 0 | 0 | 0 | 1 |
| EN1 | BDNF | 0 | 0 | 0 | 0 | 1 | 0 | 1 |
| EN1 | NCBP1 | 0 | 0 | 1 | 0 | 0 | 0 | 1 |
| EN1 | VAT1L | 0 | 0 | 1 | 0 | 0 | 0 | 1 |
| EN1 | PPFIBP1 | 0 | 0 | 1 | 0 | 0 | 0 | 1 |
| FOXJ2 | MPPED2 | 0 | 0 | 0 | 0 | 1 | 0 | 1 |
| EN1 | EPPIN | 0 | 0 | 1 | 0 | 0 | 0 | 1 |
| EN1 | SPINK1 | 0 | 0 | 1 | 0 | 0 | 0 | 1 |
| EN1 | GCNT1 | 0 | 0 | 1 | 0 | 0 | 0 | 1 |
| FOXJ2 | MECOM | 0 | 0 | 0 | 0 | 1 | 0 | 1 |
| EN1 | DGAT2L6 | 0 | 0 | 1 | 0 | 0 | 0 | 1 |
| FOXJ2 | CTXN1 | 0 | 0 | 0 | 0 | 1 | 0 | 1 |
| EN1 | PMCH | 0 | 0 | 1 | 0 | 0 | 0 | 1 |
| EN1 | DBNDD2 | 0 | 0 | 1 | 0 | 0 | 0 | 1 |
| EN1 | KLK3 | 0 | 0 | 1 | 0 | 0 | 0 | 1 |
| EN1 | CMTM7 | 0 | 0 | 1 | 0 | 0 | 0 | 1 |
| EN1 | FBXW2 | 0 | 0 | 1 | 0 | 0 | 0 | 1 |
| EN1 | USP11 | 0 | 0 | 1 | 0 | 0 | 0 | 1 |
| EN1 | AGPAT9 | 0 | 0 | 1 | 0 | 0 | 0 | 1 |
| EN1 | ZW10 | 0 | 0 | 1 | 0 | 0 | 0 | 1 |
| EN1 | PDS5B | 0 | 0 | 1 | 0 | 0 | 0 | 1 |
| FOXJ2 | C7ORF60 | 0 | 0 | 0 | 0 | 1 | 0 | 1 |
| EN1 | LHFPL5 | 0 | 0 | 1 | 0 | 0 | 0 | 1 |
| EN1 | CRISP1 | 0 | 0 | 1 | 0 | 0 | 0 | 1 |
| EN1 | SEC14L1 | 0 | 0 | 1 | 0 | 0 | 0 | 1 |
| EN1 | AMY2A | 0 | 0 | 1 | 0 | 0 | 0 | 1 |
| EN1 | TM7SF2 | 0 | 0 | 1 | 0 | 0 | 0 | 1 |
| FOXJ2 | ARL4C | 0 | 0 | 0 | 0 | 1 | 0 | 1 |
| FOXJ2 | BCL11A | 0 | 0 | 0 | 0 | 1 | 0 | 1 |
| EN1 | CAMLG | 0 | 0 | 0 | 0 | 1 | 0 | 1 |
| FOXJ2 | TBXAS1 | 0 | 0 | 0 | 0 | 1 | 0 | 1 |
| FOXJ2 | C5ORF64 | 0 | 0 | 0 | 0 | 1 | 0 | 1 |
| EN1 | NPPC | 0 | 0 | 1 | 0 | 0 | 0 | 1 |
| FOXJ2 | PLEC | 0 | 0 | 0 | 0 | 1 | 0 | 1 |
| EN1 | LGMN | 0 | 0 | 1 | 0 | 0 | 0 | 1 |
| FOXJ2 | ERRFI1 | 0 | 0 | 0 | 0 | 1 | 0 | 1 |
| FOXJ2 | CD180 | 0 | 0 | 0 | 0 | 1 | 0 | 1 |
| FOXJ2 | GRIN2B | 0 | 0 | 0 | 0 | 1 | 0 | 1 |
| EN1 | LIN37 | 0 | 0 | 1 | 0 | 0 | 0 | 1 |
| EN1 | ZNF536 | 0 | 0 | 1 | 0 | 0 | 0 | 1 |
| EN1 | HIBADH | 0 | 0 | 1 | 0 | 0 | 0 | 1 |
| EN1 | NAT14 | 0 | 0 | 1 | 0 | 0 | 0 | 1 |
| EN1 | NRG1 | 0 | 0 | 0 | 0 | 1 | 0 | 1 |
| EN1 | CHRNB1 | 0 | 0 | 1 | 0 | 0 | 0 | 1 |
| EN1 | OXR1 | 0 | 0 | 1 | 0 | 0 | 0 | 1 |
| EN1 | MRPS25 | 0 | 0 | 1 | 0 | 0 | 0 | 1 |
| EN1 | CYP39A1 | 0 | 0 | 1 | 0 | 0 | 0 | 1 |
| EN1 | PAXIP1 | 0 | 0 | 1 | 0 | 0 | 0 | 1 |
| FOXJ2 | KLF3 | 0 | 0 | 0 | 0 | 1 | 0 | 1 |
| FOXJ2 | TWIST1 | 0 | 0 | 0 | 0 | 1 | 0 | 1 |
| EN1 | SLC22A25 | 0 | 0 | 1 | 0 | 0 | 0 | 1 |
| EN1 | N4BP3 | 0 | 0 | 1 | 0 | 0 | 0 | 1 |
| EN1 | DNAJC9 | 0 | 0 | 1 | 0 | 0 | 0 | 1 |
| EN1 | HAND1 | 0 | 0 | 1 | 0 | 0 | 0 | 1 |
| EN1 | ACHE | 0 | 0 | 1 | 0 | 0 | 0 | 1 |
| EN1 | URM1 | 0 | 0 | 1 | 0 | 0 | 0 | 1 |
| EN1 | MTX3 | 0 | 0 | 1 | 0 | 0 | 0 | 1 |
| EN1 | SKA3 | 0 | 0 | 1 | 0 | 0 | 0 | 1 |
| EN1 | ADCY9 | 0 | 0 | 1 | 0 | 0 | 0 | 1 |
| EN1 | IFT88 | 0 | 0 | 1 | 0 | 0 | 0 | 1 |
| EN1 | REG1A | 0 | 0 | 1 | 0 | 0 | 0 | 1 |
| EN1 | SIPA1L1 | 0 | 0 | 0 | 0 | 1 | 0 | 1 |
| EN1 | CNTN4 | 0 | 0 | 1 | 0 | 0 | 0 | 1 |
| EN1 | OR2B6 | 0 | 0 | 1 | 0 | 0 | 0 | 1 |
| EN1 | TCF20 | 0 | 0 | 1 | 0 | 0 | 0 | 1 |
| EN1 | RAPGEF5 | 0 | 0 | 0 | 0 | 1 | 0 | 1 |
| EN1 | RNASE11 | 0 | 0 | 1 | 0 | 0 | 0 | 1 |
| FOXJ2 | TMEM258 | 0 | 0 | 0 | 0 | 1 | 0 | 1 |
| EN1 | DCAKD | 0 | 0 | 1 | 0 | 0 | 0 | 1 |
| EN1 | CCDC23 | 0 | 0 | 1 | 0 | 0 | 0 | 1 |
| SATB1 | CR2 | 0 | 0 | 0 | 0 | 0 | 1 | 1 |
| EN1 | BDH2 | 0 | 0 | 1 | 0 | 0 | 0 | 1 |
| FOXJ2 | POLG | 0 | 0 | 0 | 0 | 1 | 0 | 1 |
| EN1 | SPTLC1 | 0 | 0 | 1 | 0 | 0 | 0 | 1 |
| EN1 | TUBB2A | 0 | 0 | 1 | 0 | 0 | 0 | 1 |
| EN1 | C10orf90 | 0 | 0 | 1 | 0 | 0 | 0 | 1 |
| EN1 | PSMA8 | 0 | 0 | 1 | 0 | 0 | 0 | 1 |
| EN1 | FBXL18 | 0 | 0 | 1 | 0 | 0 | 0 | 1 |
| EN1 | SORBS2 | 0 | 0 | 0 | 0 | 1 | 0 | 1 |
| FOXJ2 | PDE4D | 0 | 0 | 0 | 0 | 1 | 0 | 1 |
| FOXJ2 | ARNTL | 0 | 0 | 0 | 0 | 1 | 0 | 1 |
| FOXJ2 | LUC7L | 0 | 0 | 0 | 0 | 1 | 0 | 1 |
| FOXJ2 | NPTX2 | 0 | 0 | 0 | 0 | 1 | 0 | 1 |
| EN1 | COL25A1 | 0 | 0 | 1 | 0 | 0 | 0 | 1 |
| EN1 | PGBD1 | 0 | 0 | 1 | 0 | 0 | 0 | 1 |
| EN1 | ISL1 | 0 | 0 | 0 | 0 | 1 | 0 | 1 |
| EN1 | TMCO5B | 0 | 0 | 1 | 0 | 0 | 0 | 1 |
| FOXJ2 | EGR2 | 0 | 0 | 0 | 0 | 1 | 0 | 1 |
| FOXJ2 | PRMT6 | 0 | 0 | 0 | 0 | 1 | 0 | 1 |
| FOXJ2 | COL10A1 | 0 | 0 | 0 | 0 | 1 | 0 | 1 |
| EN1 | TBCA | 0 | 0 | 1 | 0 | 0 | 0 | 1 |
| SATB1 | HBE1 | 0 | 0 | 0 | 0 | 0 | 1 | 1 |
| EN1 | TP53INP1 | 0 | 0 | 1 | 0 | 0 | 0 | 1 |
| FOXJ2 | SLIT3 | 0 | 0 | 0 | 0 | 1 | 0 | 1 |
| EN1 | ARPP19 | 0 | 0 | 1 | 0 | 0 | 0 | 1 |
| EN1 | ZNF236 | 0 | 0 | 1 | 0 | 0 | 0 | 1 |
| FOXJ2 | ROGDI | 0 | 0 | 0 | 0 | 1 | 0 | 1 |
| FOXJ2 | ANP32D | 0 | 0 | 0 | 0 | 1 | 0 | 1 |
| EN1 | LIPC | 0 | 0 | 1 | 0 | 0 | 0 | 1 |
| EN1 | PARP14 | 0 | 0 | 1 | 0 | 0 | 0 | 1 |
| FOXJ2 | CHCHD7 | 0 | 0 | 0 | 0 | 1 | 0 | 1 |
| EN1 | AIFM3 | 0 | 0 | 1 | 0 | 0 | 0 | 1 |
| FOXJ2 | SYT6 | 0 | 0 | 0 | 0 | 1 | 0 | 1 |
| EN1 | FUT9 | 0 | 0 | 1 | 0 | 0 | 0 | 1 |
| EN1 | MEGF6 | 0 | 0 | 1 | 0 | 0 | 0 | 1 |
| EN1 | POLE2 | 0 | 0 | 1 | 0 | 0 | 0 | 1 |
| EN1 | KCNT1 | 0 | 0 | 1 | 0 | 0 | 0 | 1 |
| EN1 | OBFC1 | 0 | 0 | 1 | 0 | 0 | 0 | 1 |
| FOXJ2 | NRAS | 0 | 0 | 0 | 0 | 1 | 0 | 1 |
| FOXJ2 | MGLL | 0 | 0 | 0 | 0 | 1 | 0 | 1 |
| EN1 | HAO2 | 0 | 0 | 1 | 0 | 0 | 0 | 1 |
| EN1 | BGLAP | 0 | 0 | 1 | 0 | 0 | 0 | 1 |
| EN1 | FBXO24 | 0 | 0 | 1 | 0 | 0 | 0 | 1 |
| FOXJ2 | C17ORF47 | 0 | 0 | 0 | 0 | 1 | 0 | 1 |
| EN1 | MTRF1L | 0 | 0 | 1 | 0 | 0 | 0 | 1 |
| EN1 | MED23 | 0 | 0 | 1 | 0 | 0 | 0 | 1 |
| FOXJ2 | ARTN | 0 | 0 | 0 | 0 | 1 | 0 | 1 |
| FOXJ2 | HOXA11 | 0 | 0 | 0 | 0 | 1 | 0 | 1 |
| EN1 | SCYL3 | 0 | 0 | 1 | 0 | 0 | 0 | 1 |
| EN1 | MOS | 0 | 0 | 1 | 0 | 0 | 0 | 1 |
| EN1 | TMEM143 | 0 | 0 | 1 | 0 | 0 | 0 | 1 |
| FOXJ2 | ELMO3 | 0 | 0 | 0 | 0 | 1 | 0 | 1 |
| EN1 | NABP2 | 0 | 0 | 1 | 0 | 0 | 0 | 1 |
| EN1 | FFAR2 | 0 | 0 | 1 | 0 | 0 | 0 | 1 |
| SATB1 | IL2RA | 0 | 0 | 0 | 0 | 0 | 1 | 1 |
| EN1 | PIK3CG | 0 | 0 | 1 | 0 | 0 | 0 | 1 |
| EN1 | F8 | 0 | 0 | 1 | 0 | 0 | 0 | 1 |
| FOXJ2 | PKIA | 0 | 0 | 0 | 0 | 1 | 0 | 1 |
| FOXJ2 | TWIST2 | 0 | 0 | 0 | 0 | 1 | 0 | 1 |
| EN1 | STOML1 | 0 | 0 | 1 | 0 | 0 | 0 | 1 |
| EN1 | TOP3A | 0 | 0 | 1 | 0 | 0 | 0 | 1 |
| EN1 | LRRC8B | 0 | 0 | 1 | 0 | 0 | 0 | 1 |
| EN1 | SH3BGRL2 | 0 | 0 | 0 | 0 | 1 | 0 | 1 |
| EN1 | SLC2A7 | 0 | 0 | 1 | 0 | 0 | 0 | 1 |
| EN1 | PIGO | 0 | 0 | 1 | 0 | 0 | 0 | 1 |
| FOXJ2 | PLAG1 | 0 | 0 | 0 | 0 | 1 | 0 | 1 |
| FOXJ2 | FGF13 | 0 | 0 | 0 | 0 | 1 | 0 | 1 |
| EN1 | VKORC1 | 0 | 0 | 1 | 0 | 0 | 0 | 1 |
| FOXJ2 | IFFO1 | 0 | 0 | 0 | 0 | 1 | 0 | 1 |
| EN1 | GALNT11 | 0 | 0 | 1 | 0 | 0 | 0 | 1 |
| EN1 | PLA2G12B | 0 | 0 | 1 | 0 | 0 | 0 | 1 |
| EN1 | TAF1 | 0 | 0 | 1 | 0 | 0 | 0 | 1 |
| FOXJ2 | SERINC5 | 0 | 0 | 0 | 0 | 1 | 0 | 1 |
| EN1 | EXOSC1 | 0 | 0 | 1 | 0 | 0 | 0 | 1 |
| FOXJ2 | NEUROD2 | 0 | 0 | 0 | 0 | 1 | 0 | 1 |
| EN1 | SNORD12 | 0 | 0 | 1 | 0 | 0 | 0 | 1 |
| FOXJ2 | HESX1 | 0 | 0 | 0 | 0 | 1 | 0 | 1 |
| EN1 | CEBPA | 0 | 0 | 1 | 0 | 0 | 0 | 1 |
| FOXJ2 | RAB5B | 0 | 0 | 0 | 0 | 1 | 0 | 1 |
| EN1 | PIK3CD | 0 | 0 | 1 | 0 | 0 | 0 | 1 |
| EN1 | NEURL3 | 0 | 0 | 1 | 0 | 0 | 0 | 1 |
| EN1 | AIFM1 | 0 | 0 | 1 | 0 | 0 | 0 | 1 |
| EN1 | NPRL3 | 0 | 0 | 1 | 0 | 0 | 0 | 1 |
| FOXJ2 | FOXN3 | 0 | 0 | 0 | 0 | 1 | 0 | 1 |
| EN1 | PLEKHS1 | 0 | 0 | 1 | 0 | 0 | 0 | 1 |
| EN1 | MIR7-2 | 0 | 0 | 1 | 0 | 0 | 0 | 1 |
| EN1 | SHPK | 0 | 0 | 1 | 0 | 0 | 0 | 1 |
| FOXJ2 | MYOCD | 0 | 0 | 0 | 0 | 1 | 0 | 1 |
| EN1 | ZNF704 | 0 | 0 | 1 | 0 | 0 | 0 | 1 |
| EN1 | PRICKLE2 | 0 | 0 | 1 | 0 | 0 | 0 | 1 |
| EN1 | PRSS35 | 0 | 0 | 1 | 0 | 0 | 0 | 1 |
| EN1 | OTOP3 | 0 | 0 | 1 | 0 | 0 | 0 | 1 |
| EN1 | ART4 | 0 | 0 | 1 | 0 | 0 | 0 | 1 |
| EN1 | NPC2 | 0 | 0 | 1 | 0 | 0 | 0 | 1 |
| EN1 | ANKRD40 | 0 | 0 | 1 | 0 | 0 | 0 | 1 |
| EN1 | ITIH5 | 0 | 0 | 1 | 0 | 0 | 0 | 1 |
| EN1 | ADAMTS6 | 0 | 0 | 1 | 0 | 0 | 0 | 1 |
| EN1 | PUM2 | 0 | 0 | 1 | 0 | 0 | 0 | 1 |
| EN1 | CD300E | 0 | 0 | 1 | 0 | 0 | 0 | 1 |
| EN1 | OR52A1 | 0 | 0 | 1 | 0 | 0 | 0 | 1 |
| EN1 | NAT9 | 0 | 0 | 1 | 0 | 0 | 0 | 1 |
| EN1 | FGF17 | 0 | 0 | 0 | 0 | 1 | 0 | 1 |
| EN1 | ZADH2 | 0 | 0 | 0 | 0 | 1 | 0 | 1 |
| FOXJ2 | CADM1 | 0 | 0 | 0 | 0 | 1 | 0 | 1 |
| FOXJ2 | CDK6 | 0 | 0 | 0 | 0 | 1 | 0 | 1 |
| EN1 | VDAC3 | 0 | 0 | 1 | 0 | 0 | 0 | 1 |
| EN1 | FUT11 | 0 | 0 | 1 | 0 | 0 | 0 | 1 |
| EN1 | CDH22 | 0 | 0 | 1 | 0 | 0 | 0 | 1 |
| EN1 | PTPRZ1 | 0 | 0 | 1 | 0 | 0 | 0 | 1 |
| FOXJ2 | GFI1 | 0 | 0 | 0 | 0 | 1 | 0 | 1 |
| EN1 | SNORA21 | 0 | 0 | 1 | 0 | 0 | 0 | 1 |
| EN1 | PTBP2 | 0 | 0 | 1 | 0 | 0 | 0 | 1 |
| EN1 | RNF165 | 0 | 0 | 1 | 0 | 0 | 0 | 1 |
| EN1 | CHAF1B | 0 | 0 | 1 | 0 | 0 | 0 | 1 |
| EN1 | KANSL2 | 0 | 0 | 1 | 0 | 0 | 0 | 1 |
| EN1 | CXCL9 | 0 | 0 | 1 | 0 | 0 | 0 | 1 |
| EN1 | PPFIA1 | 0 | 0 | 1 | 0 | 0 | 0 | 1 |
| EN1 | CYP7A1 | 0 | 0 | 1 | 0 | 0 | 0 | 1 |
| EN1 | RIMKLB | 0 | 0 | 1 | 0 | 0 | 0 | 1 |
| EN1 | MAP4K4 | 0 | 0 | 0 | 0 | 1 | 0 | 1 |
| EN1 | OR2T8 | 0 | 0 | 1 | 0 | 0 | 0 | 1 |
| EN1 | ETFDH | 0 | 0 | 1 | 0 | 0 | 0 | 1 |
| EN1 | MGME1 | 0 | 0 | 1 | 0 | 0 | 0 | 1 |
| FOXJ2 | HOXC6 | 0 | 0 | 0 | 0 | 1 | 0 | 1 |
| EN1 | MORF4L2 | 0 | 0 | 0 | 0 | 1 | 0 | 1 |
| EN1 | MBNL1 | 0 | 0 | 1 | 0 | 0 | 0 | 1 |
| EN1 | OR56B1 | 0 | 0 | 1 | 0 | 0 | 0 | 1 |
| FOXJ2 | DRD3 | 0 | 0 | 0 | 0 | 1 | 0 | 1 |
| EN1 | SOX10 | 0 | 0 | 1 | 0 | 0 | 0 | 1 |
| FOXJ2 | CDH9 | 0 | 0 | 0 | 0 | 1 | 0 | 1 |
| FOXJ2 | ANGEL1 | 0 | 0 | 0 | 0 | 1 | 0 | 1 |
| EN1 | TRPV2 | 0 | 0 | 1 | 0 | 0 | 0 | 1 |
| FOXJ2 | HNF4A | 0 | 0 | 0 | 0 | 1 | 0 | 1 |
| EN1 | MRPS21 | 0 | 0 | 1 | 0 | 0 | 0 | 1 |
| FOXJ2 | LINC00474 | 0 | 0 | 0 | 0 | 1 | 0 | 1 |
| EN1 | CPVL | 0 | 0 | 1 | 0 | 0 | 0 | 1 |
| FOXJ2 | PNOC | 0 | 0 | 0 | 0 | 1 | 0 | 1 |
| EN1 | USP35 | 0 | 0 | 1 | 0 | 0 | 0 | 1 |
| EN1 | IMPG1 | 0 | 0 | 1 | 0 | 0 | 0 | 1 |
| EN1 | TPM3 | 0 | 0 | 1 | 0 | 0 | 0 | 1 |
| FOXJ2 | AP2M1 | 0 | 0 | 0 | 0 | 1 | 0 | 1 |
| SATB1 | CYBB | 0 | 0 | 0 | 0 | 0 | 1 | 1 |
| EN1 | CTDSP2 | 0 | 0 | 1 | 0 | 0 | 0 | 1 |
| FOXJ2 | DCHS1 | 0 | 0 | 0 | 0 | 1 | 0 | 1 |
| EN1 | ECI1 | 0 | 0 | 1 | 0 | 0 | 0 | 1 |
| FOXJ2 | ATP2A2 | 0 | 0 | 0 | 0 | 1 | 0 | 1 |
| EN1 | PLEKHG3 | 0 | 0 | 1 | 0 | 0 | 0 | 1 |
| EN1 | CNGB1 | 0 | 0 | 1 | 0 | 0 | 0 | 1 |
| EN1 | HSF4 | 0 | 0 | 1 | 0 | 0 | 0 | 1 |
| EN1 | P2RY12 | 0 | 0 | 1 | 0 | 0 | 0 | 1 |
| EN1 | PSMD10 | 0 | 0 | 1 | 0 | 0 | 0 | 1 |
| EN1 | RORB | 0 | 0 | 1 | 0 | 0 | 0 | 1 |
| EN1 | KATNBL1 | 0 | 0 | 1 | 0 | 0 | 0 | 1 |
| EN1 | DCAF11 | 0 | 0 | 1 | 0 | 0 | 0 | 1 |
| FOXJ2 | PRDM12 | 0 | 0 | 0 | 0 | 1 | 0 | 1 |
| EN1 | DUSP22 | 0 | 0 | 1 | 0 | 0 | 0 | 1 |
| EN1 | DCUN1D5 | 0 | 0 | 1 | 0 | 0 | 0 | 1 |
| EN1 | PLEK | 0 | 0 | 1 | 0 | 0 | 0 | 1 |
| EN1 | KLRD1 | 0 | 0 | 1 | 0 | 0 | 0 | 1 |
| EN1 | TSHB | 0 | 0 | 1 | 0 | 0 | 0 | 1 |
| EN1 | IFIT1 | 0 | 0 | 1 | 0 | 0 | 0 | 1 |
| FOXJ2 | IPO4 | 0 | 0 | 0 | 0 | 1 | 0 | 1 |
| EN1 | ASB3 | 0 | 0 | 1 | 0 | 0 | 0 | 1 |
| EN1 | LAMA4 | 0 | 0 | 1 | 0 | 0 | 0 | 1 |
| EN1 | ARF5 | 0 | 0 | 1 | 0 | 0 | 0 | 1 |
| FOXJ2 | PRKAG1 | 0 | 0 | 0 | 0 | 1 | 0 | 1 |
| EN1 | SLC9A9 | 0 | 0 | 1 | 0 | 0 | 0 | 1 |
| EN1 | C6orf222 | 0 | 0 | 1 | 0 | 0 | 0 | 1 |
| EN1 | LCE1D | 0 | 0 | 1 | 0 | 0 | 0 | 1 |
| EN1 | MRPL1 | 0 | 0 | 1 | 0 | 0 | 0 | 1 |
| EN1 | CLDN10 | 0 | 0 | 1 | 0 | 0 | 0 | 1 |
| FOXJ2 | NFIB | 0 | 0 | 0 | 0 | 1 | 0 | 1 |
| FOXJ2 | PPM1B | 0 | 0 | 0 | 0 | 1 | 0 | 1 |
| FOXJ2 | SOX6 | 0 | 0 | 0 | 0 | 1 | 0 | 1 |
| FOXJ2 | HOXC12 | 0 | 0 | 0 | 0 | 1 | 0 | 1 |
| EN1 | DNMT1 | 0 | 0 | 1 | 0 | 0 | 0 | 1 |
| EN1 | PPP1R18 | 0 | 0 | 1 | 0 | 0 | 0 | 1 |
| EN1 | TAMM41 | 0 | 0 | 1 | 0 | 0 | 0 | 1 |
| EN1 | BLCAP | 0 | 0 | 1 | 0 | 0 | 0 | 1 |
| EN1 | FAM53C | 0 | 0 | 0 | 0 | 1 | 0 | 1 |
| FOXJ2 | C6ORF62 | 0 | 0 | 0 | 0 | 1 | 0 | 1 |
| FOXJ2 | FGF7 | 0 | 0 | 0 | 0 | 1 | 0 | 1 |
| EN1 | TARS2 | 0 | 0 | 1 | 0 | 0 | 0 | 1 |
| FOXJ2 | ZC3H6 | 0 | 0 | 0 | 0 | 1 | 0 | 1 |
| EN1 | GREM1 | 0 | 0 | 0 | 0 | 1 | 0 | 1 |
| EN1 | CAPN1 | 0 | 0 | 1 | 0 | 0 | 0 | 1 |
| EN1 | TFEB | 0 | 0 | 0 | 0 | 1 | 0 | 1 |
| FOXJ2 | TMEM156 | 0 | 0 | 0 | 0 | 1 | 0 | 1 |
| EN1 | SDK2 | 0 | 0 | 1 | 0 | 0 | 0 | 1 |
| EN1 | ARHGEF10 | 0 | 0 | 1 | 0 | 0 | 0 | 1 |
| EN1 | MIR126 | 0 | 0 | 1 | 0 | 0 | 0 | 1 |
| FOXJ2 | SUPT4H1 | 0 | 0 | 0 | 0 | 1 | 0 | 1 |
| EN1 | FAM83E | 0 | 0 | 1 | 0 | 0 | 0 | 1 |
| FOXJ2 | CCDC109B | 0 | 0 | 0 | 0 | 1 | 0 | 1 |
| EN1 | PITHD1 | 0 | 0 | 1 | 0 | 0 | 0 | 1 |
| EN1 | C3orf62 | 0 | 0 | 1 | 0 | 0 | 0 | 1 |
| EN1 | DUS2 | 0 | 0 | 1 | 0 | 0 | 0 | 1 |
| EN1 | EMP3 | 0 | 0 | 1 | 0 | 0 | 0 | 1 |
| FOXJ2 | NCDN | 0 | 0 | 0 | 0 | 1 | 0 | 1 |
| FOXJ2 | CELF4 | 0 | 0 | 0 | 0 | 1 | 0 | 1 |
| EN1 | CDCA5 | 0 | 0 | 1 | 0 | 0 | 0 | 1 |
| EN1 | PTPN22 | 0 | 0 | 1 | 0 | 0 | 0 | 1 |
| FOXJ2 | IFNA17 | 0 | 0 | 0 | 0 | 1 | 0 | 1 |
| FOXJ2 | POLR3F | 0 | 0 | 0 | 0 | 1 | 0 | 1 |
| EN1 | RCAN1 | 0 | 0 | 1 | 0 | 0 | 0 | 1 |
| EN1 | TRIB3 | 0 | 0 | 1 | 0 | 0 | 0 | 1 |
| EN1 | ROPN1L | 0 | 0 | 1 | 0 | 0 | 0 | 1 |
| FOXJ2 | CASC4 | 0 | 0 | 0 | 0 | 1 | 0 | 1 |
| EN1 | HES2 | 0 | 0 | 1 | 0 | 0 | 0 | 1 |
| FOXJ2 | FOXP2 | 0 | 0 | 0 | 0 | 1 | 0 | 1 |
| FOXJ2 | TRIM2 | 0 | 0 | 0 | 0 | 1 | 0 | 1 |
| FOXJ2 | YRDC | 0 | 0 | 0 | 0 | 1 | 0 | 1 |
| FOXJ2 | PTHLH | 0 | 0 | 0 | 0 | 1 | 0 | 1 |
| EN1 | GALNT10 | 0 | 0 | 1 | 0 | 0 | 0 | 1 |
| EN1 | ARL9 | 0 | 0 | 1 | 0 | 0 | 0 | 1 |
| SATB1 | GATA3 | 0 | 0 | 0 | 0 | 0 | 1 | 1 |
| FOXJ2 | UCKL1 | 0 | 0 | 0 | 0 | 1 | 0 | 1 |
| EN1 | SLC23A3 | 0 | 0 | 1 | 0 | 0 | 0 | 1 |
| EN1 | USP49 | 0 | 0 | 1 | 0 | 0 | 0 | 1 |
| FOXJ2 | ACACA | 0 | 0 | 0 | 0 | 1 | 0 | 1 |
| FOXJ2 | IRS4 | 0 | 0 | 0 | 0 | 1 | 0 | 1 |
| FOXJ2 | ZC3H14 | 0 | 0 | 0 | 0 | 1 | 0 | 1 |
| EN1 | NEU2 | 0 | 0 | 1 | 0 | 0 | 0 | 1 |
| EN1 | PEX2 | 0 | 0 | 0 | 0 | 1 | 0 | 1 |
| EN1 | ADM2 | 0 | 0 | 1 | 0 | 0 | 0 | 1 |
| FOXJ2 | UTRN | 0 | 0 | 0 | 0 | 1 | 0 | 1 |
| EN1 | C8G | 0 | 0 | 1 | 0 | 0 | 0 | 1 |
| EN1 | KIAA1549 | 0 | 0 | 1 | 0 | 0 | 0 | 1 |
| EN1 | MAP3K15 | 0 | 0 | 1 | 0 | 0 | 0 | 1 |
| FOXJ2 | MAP2 | 0 | 0 | 0 | 0 | 1 | 0 | 1 |
| EN1 | RC3H1 | 0 | 0 | 1 | 0 | 0 | 0 | 1 |
| EN1 | CREM | 0 | 0 | 0 | 0 | 1 | 0 | 1 |
| EN1 | RAD51AP1 | 0 | 0 | 1 | 0 | 0 | 0 | 1 |
| FOXJ2 | BUB3 | 0 | 0 | 0 | 0 | 1 | 0 | 1 |
| EN1 | MIR140 | 0 | 0 | 1 | 0 | 0 | 0 | 1 |
| EN1 | SLC25A22 | 0 | 0 | 1 | 0 | 0 | 0 | 1 |
| EN1 | HAGH | 0 | 0 | 1 | 0 | 0 | 0 | 1 |
| SATB1 | BCL2 | 0 | 0 | 0 | 0 | 0 | 1 | 1 |
| FOXJ2 | TUBA1A | 0 | 0 | 0 | 0 | 1 | 0 | 1 |
| EN1 | C5orf28 | 0 | 0 | 1 | 0 | 0 | 0 | 1 |
| FOXJ2 | RORB | 0 | 0 | 0 | 0 | 1 | 0 | 1 |
| EN1 | EDA | 0 | 0 | 1 | 0 | 0 | 0 | 1 |
| FOXJ2 | TRPS1 | 0 | 0 | 0 | 0 | 1 | 0 | 1 |
| EN1 | GJB3 | 0 | 0 | 1 | 0 | 0 | 0 | 1 |
| EN1 | SOCS1 | 0 | 0 | 1 | 0 | 0 | 0 | 1 |
| FOXJ2 | DAAM1 | 0 | 0 | 0 | 0 | 1 | 0 | 1 |
| FOXJ2 | HDAC9 | 0 | 0 | 0 | 0 | 1 | 0 | 1 |
| EN1 | DMC1 | 0 | 0 | 1 | 0 | 0 | 0 | 1 |
| EN1 | F7 | 0 | 0 | 1 | 0 | 0 | 0 | 1 |
| EN1 | RREB1 | 0 | 0 | 1 | 0 | 0 | 0 | 1 |
| EN1 | PDE4B | 0 | 0 | 1 | 0 | 0 | 0 | 1 |
| FOXJ2 | CRH | 0 | 0 | 0 | 0 | 1 | 0 | 1 |
| EN1 | MAMSTR | 0 | 0 | 1 | 0 | 0 | 0 | 1 |
| FOXJ2 | HMCN1 | 0 | 0 | 0 | 0 | 1 | 0 | 1 |
| FOXJ2 | FRAS1 | 0 | 0 | 0 | 0 | 1 | 0 | 1 |
| FOXJ2 | GHR | 0 | 0 | 0 | 0 | 1 | 0 | 1 |
| EN1 | OR2D3 | 0 | 0 | 1 | 0 | 0 | 0 | 1 |
| EN1 | PIGM | 0 | 0 | 1 | 0 | 0 | 0 | 1 |
| EN1 | NOL6 | 0 | 0 | 1 | 0 | 0 | 0 | 1 |
| EN1 | ARF4 | 0 | 0 | 1 | 0 | 0 | 0 | 1 |
| EN1 | AS3MT | 0 | 0 | 1 | 0 | 0 | 0 | 1 |
| FOXJ2 | HAS2 | 0 | 0 | 0 | 0 | 1 | 0 | 1 |
| FOXJ2 | RBM39 | 0 | 0 | 0 | 0 | 1 | 0 | 1 |
| EN1 | UCHL1 | 0 | 0 | 1 | 0 | 0 | 0 | 1 |
| FOXJ2 | C1ORF122 | 0 | 0 | 0 | 0 | 1 | 0 | 1 |
| EN1 | MYOG | 0 | 0 | 0 | 0 | 1 | 0 | 1 |
| FOXJ2 | CUX1 | 0 | 0 | 0 | 0 | 1 | 0 | 1 |
| EN1 | COPS3 | 0 | 0 | 1 | 0 | 0 | 0 | 1 |
| EN1 | IL23A | 0 | 0 | 1 | 0 | 0 | 0 | 1 |
| EN1 | WDR73 | 0 | 0 | 1 | 0 | 0 | 0 | 1 |
| EN1 | IL22 | 0 | 0 | 1 | 0 | 0 | 0 | 1 |
| FOXJ2 | SLC34A3 | 0 | 0 | 0 | 0 | 1 | 0 | 1 |
| EN1 | DUSP21 | 0 | 0 | 1 | 0 | 0 | 0 | 1 |
| EN1 | BRSK1 | 0 | 0 | 1 | 0 | 0 | 0 | 1 |
| EN1 | MYL1 | 0 | 0 | 1 | 0 | 0 | 0 | 1 |
| EN1 | MEIS2 | 0 | 0 | 1 | 0 | 0 | 0 | 1 |
| EN1 | SNX19 | 0 | 0 | 1 | 0 | 0 | 0 | 1 |
| EN1 | NTRK2 | 0 | 0 | 1 | 0 | 0 | 0 | 1 |
| EN1 | CCDC28B | 0 | 0 | 1 | 0 | 0 | 0 | 1 |
| EN1 | LSM5 | 0 | 0 | 1 | 0 | 0 | 0 | 1 |
| FOXJ2 | ARF6 | 0 | 0 | 0 | 0 | 1 | 0 | 1 |
| EN1 | C21orf91 | 0 | 0 | 1 | 0 | 0 | 0 | 1 |
| EN1 | UNC45B | 0 | 0 | 1 | 0 | 0 | 0 | 1 |
| EN1 | DOCK9 | 0 | 0 | 1 | 0 | 0 | 0 | 1 |
| FOXJ2 | MSL3 | 0 | 0 | 0 | 0 | 1 | 0 | 1 |
| EN1 | EID1 | 0 | 0 | 1 | 0 | 0 | 0 | 1 |
| EN1 | MRPL27 | 0 | 0 | 1 | 0 | 0 | 0 | 1 |
| FOXJ2 | STX5 | 0 | 0 | 0 | 0 | 1 | 0 | 1 |
| FOXJ2 | DNAH12 | 0 | 0 | 0 | 0 | 1 | 0 | 1 |
| FOXJ2 | SIPA1 | 0 | 0 | 0 | 0 | 1 | 0 | 1 |
| FOXJ2 | RARB | 0 | 0 | 0 | 0 | 1 | 0 | 1 |
| FOXJ2 | ESRRG | 0 | 0 | 0 | 0 | 1 | 0 | 1 |
| EN1 | ARL4C | 0 | 0 | 0 | 0 | 1 | 0 | 1 |
| EN1 | SLC35A5 | 0 | 0 | 1 | 0 | 0 | 0 | 1 |
| EN1 | HVCN1 | 0 | 0 | 1 | 0 | 0 | 0 | 1 |
| FOXJ2 | NR2F1 | 0 | 0 | 0 | 0 | 1 | 0 | 1 |
| EN1 | GML | 0 | 0 | 1 | 0 | 0 | 0 | 1 |
| EN1 | JUNB | 0 | 0 | 1 | 0 | 0 | 0 | 1 |
| EN1 | MIR190B | 0 | 0 | 1 | 0 | 0 | 0 | 1 |
| EN1 | LOC101928761 | 0 | 0 | 1 | 0 | 0 | 0 | 1 |
| EN1 | PDZD2 | 0 | 0 | 0 | 0 | 1 | 0 | 1 |
| FOXJ2 | NRF1 | 0 | 0 | 0 | 0 | 1 | 0 | 1 |
| EN1 | PCNP | 0 | 0 | 1 | 0 | 0 | 0 | 1 |
| EN1 | SFSWAP | 0 | 0 | 1 | 0 | 0 | 0 | 1 |
| EN1 | GRB10 | 0 | 0 | 1 | 0 | 0 | 0 | 1 |
| EN1 | MORN1 | 0 | 0 | 1 | 0 | 0 | 0 | 1 |
| FOXJ2 | PHF20L1 | 0 | 0 | 0 | 0 | 1 | 0 | 1 |
| EN1 | CALM3 | 0 | 0 | 1 | 0 | 0 | 0 | 1 |
| EN1 | MCUR1 | 0 | 0 | 1 | 0 | 0 | 0 | 1 |
| EN1 | CEP350 | 0 | 0 | 1 | 0 | 0 | 0 | 1 |
| EN1 | GABARAPL2 | 0 | 0 | 1 | 0 | 0 | 0 | 1 |
| FOXJ2 | PNKD | 0 | 0 | 0 | 0 | 1 | 0 | 1 |
| EN1 | SEMA3G | 0 | 0 | 1 | 0 | 0 | 0 | 1 |
| EN1 | SH3GL2 | 0 | 0 | 1 | 0 | 0 | 0 | 1 |
| EN1 | PUS1 | 0 | 0 | 1 | 0 | 0 | 0 | 1 |
| FOXJ2 | NREP | 0 | 0 | 0 | 0 | 1 | 0 | 1 |
| FOXJ2 | PAFAH1B1 | 0 | 0 | 0 | 0 | 1 | 0 | 1 |
| EN1 | TGFBR3 | 0 | 0 | 1 | 0 | 0 | 0 | 1 |
| EN1 | GPC4 | 0 | 0 | 0 | 0 | 1 | 0 | 1 |
| EN1 | RNF43 | 0 | 0 | 0 | 0 | 1 | 0 | 1 |
| EN1 | PRCC | 0 | 0 | 1 | 0 | 0 | 0 | 1 |
| EN1 | C1QC | 0 | 0 | 1 | 0 | 0 | 0 | 1 |
| EN1 | MSL3P1 | 0 | 0 | 1 | 0 | 0 | 0 | 1 |
| EN1 | HMGCLL1 | 0 | 0 | 1 | 0 | 0 | 0 | 1 |
| EN1 | UTY | 0 | 0 | 0 | 0 | 1 | 0 | 1 |
| FOXJ2 | SLCO1A2 | 0 | 0 | 0 | 0 | 1 | 0 | 1 |
| EN1 | CTDNEP1 | 0 | 0 | 1 | 0 | 0 | 0 | 1 |
| FOXJ2 | HAND2 | 0 | 0 | 0 | 0 | 1 | 0 | 1 |
| EN1 | GPR142 | 0 | 0 | 1 | 0 | 0 | 0 | 1 |
| EN1 | NQO1 | 0 | 0 | 1 | 0 | 0 | 0 | 1 |
| EN1 | RASA2 | 0 | 0 | 1 | 0 | 0 | 0 | 1 |
| FOXJ2 | BCL6 | 0 | 0 | 0 | 0 | 1 | 0 | 1 |
| EN1 | NPAS4 | 0 | 0 | 1 | 0 | 0 | 0 | 1 |
| EN1 | GPC3 | 0 | 0 | 0 | 0 | 1 | 0 | 1 |
| EN1 | TDRD1 | 0 | 0 | 1 | 0 | 0 | 0 | 1 |
| FOXJ2 | CSMD3 | 0 | 0 | 0 | 0 | 1 | 0 | 1 |
| EN1 | PCDHB16 | 0 | 0 | 1 | 0 | 0 | 0 | 1 |
| FOXJ2 | ACVR1C | 0 | 0 | 0 | 0 | 1 | 0 | 1 |
| EN1 | HOXA4 | 0 | 0 | 0 | 0 | 1 | 0 | 1 |
| FOXJ2 | BMPR2 | 0 | 0 | 0 | 0 | 1 | 0 | 1 |
| FOXJ2 | ZHX2 | 0 | 0 | 0 | 0 | 1 | 0 | 1 |
| FOXJ2 | ECT2 | 0 | 0 | 0 | 0 | 1 | 0 | 1 |
| EN1 | CACNG7 | 0 | 0 | 1 | 0 | 0 | 0 | 1 |
| EN1 | PEX12 | 0 | 0 | 1 | 0 | 0 | 0 | 1 |
| FOXJ2 | SERPINI2 | 0 | 0 | 0 | 0 | 1 | 0 | 1 |
| EN1 | SYNJ2BP | 0 | 0 | 0 | 0 | 1 | 0 | 1 |
| EN1 | OSR1 | 0 | 0 | 0 | 0 | 1 | 0 | 1 |
| EN1 | C10orf82 | 0 | 0 | 1 | 0 | 0 | 0 | 1 |
| FOXJ2 | CSRNP3 | 0 | 0 | 0 | 0 | 1 | 0 | 1 |
| EN1 | TSSK2 | 0 | 0 | 1 | 0 | 0 | 0 | 1 |
| EN1 | NGB | 0 | 0 | 1 | 0 | 0 | 0 | 1 |
| EN1 | FXYD1 | 0 | 0 | 1 | 0 | 0 | 0 | 1 |
| FOXJ2 | COLCA1 | 0 | 0 | 0 | 0 | 1 | 0 | 1 |
| FOXJ2 | XPOT | 0 | 0 | 0 | 0 | 1 | 0 | 1 |
| EN1 | TNFRSF13C | 0 | 0 | 1 | 0 | 0 | 0 | 1 |
| EN1 | PPP3CB | 0 | 0 | 1 | 0 | 0 | 0 | 1 |
| EN1 | LOC285423 | 0 | 0 | 1 | 0 | 0 | 0 | 1 |
| FOXJ2 | BCL11B | 0 | 0 | 0 | 0 | 1 | 0 | 1 |
| EN1 | NREP | 0 | 0 | 1 | 0 | 0 | 0 | 1 |
| EN1 | GDF6 | 0 | 0 | 1 | 0 | 0 | 0 | 1 |
| EN1 | NOS1 | 0 | 0 | 0 | 0 | 1 | 0 | 1 |
| FOXJ2 | CDAN1 | 0 | 0 | 0 | 0 | 1 | 0 | 1 |
| EN1 | WDR17 | 0 | 0 | 1 | 0 | 0 | 0 | 1 |
| EN1 | KLF13 | 0 | 0 | 1 | 0 | 0 | 0 | 1 |
| EN1 | OXGR1 | 0 | 0 | 1 | 0 | 0 | 0 | 1 |
| EN1 | ALDOC | 0 | 0 | 1 | 0 | 0 | 0 | 1 |
| FOXJ2 | RGAG1 | 0 | 0 | 0 | 0 | 1 | 0 | 1 |
| FOXJ2 | CAST | 0 | 0 | 0 | 0 | 1 | 0 | 1 |
| EN1 | CLRN1 | 0 | 0 | 1 | 0 | 0 | 0 | 1 |
| EN1 | DGKZ | 0 | 0 | 1 | 0 | 0 | 0 | 1 |
| FOXJ2 | BAMBI | 0 | 0 | 0 | 0 | 1 | 0 | 1 |
| EN1 | ZNF239 | 0 | 0 | 1 | 0 | 0 | 0 | 1 |
| EN1 | PRSS48 | 0 | 0 | 1 | 0 | 0 | 0 | 1 |
| EN1 | ADAMTS20 | 0 | 0 | 1 | 0 | 0 | 0 | 1 |
| EN1 | OR9A2 | 0 | 0 | 1 | 0 | 0 | 0 | 1 |
| EN1 | SULF2 | 0 | 0 | 1 | 0 | 0 | 0 | 1 |
| EN1 | HIST1H2AI | 0 | 0 | 1 | 0 | 0 | 0 | 1 |
| EN1 | CISD2 | 0 | 0 | 1 | 0 | 0 | 0 | 1 |
| EN1 | DCDC5 | 0 | 0 | 0 | 0 | 1 | 0 | 1 |
| EN1 | MINPP1 | 0 | 0 | 1 | 0 | 0 | 0 | 1 |
| EN1 | TUBE1 | 0 | 0 | 1 | 0 | 0 | 0 | 1 |
| EN1 | GAK | 0 | 0 | 1 | 0 | 0 | 0 | 1 |
| EN1 | ZNF483 | 0 | 0 | 1 | 0 | 0 | 0 | 1 |
| EN1 | NYX | 0 | 0 | 1 | 0 | 0 | 0 | 1 |
| EN1 | AKR1D1 | 0 | 0 | 1 | 0 | 0 | 0 | 1 |
| EN1 | FAM20C | 0 | 0 | 1 | 0 | 0 | 0 | 1 |
| EN1 | MPC1 | 0 | 0 | 1 | 0 | 0 | 0 | 1 |
| FOXJ2 | ID1 | 0 | 0 | 0 | 0 | 1 | 0 | 1 |
| FOXJ2 | HSD11B1 | 0 | 0 | 0 | 0 | 1 | 0 | 1 |
| EN1 | KLHL35 | 0 | 0 | 1 | 0 | 0 | 0 | 1 |
| EN1 | ACTR3 | 0 | 0 | 1 | 0 | 0 | 0 | 1 |
| EN1 | PIGP | 0 | 0 | 1 | 0 | 0 | 0 | 1 |
| EN1 | FCRL6 | 0 | 0 | 1 | 0 | 0 | 0 | 1 |
| EN1 | FSCB | 0 | 0 | 1 | 0 | 0 | 0 | 1 |
| FOXJ2 | PDZD9 | 0 | 0 | 0 | 0 | 1 | 0 | 1 |
| EN1 | SLC2A12 | 0 | 0 | 1 | 0 | 0 | 0 | 1 |
| FOXJ2 | ERG | 0 | 0 | 0 | 0 | 1 | 0 | 1 |
| EN1 | CGRRF1 | 0 | 0 | 1 | 0 | 0 | 0 | 1 |
| FOXJ2 | ZNF423 | 0 | 0 | 0 | 0 | 1 | 0 | 1 |
| FOXJ2 | FAM26E | 0 | 0 | 0 | 0 | 1 | 0 | 1 |
| EN1 | TCF4 | 0 | 0 | 0 | 0 | 1 | 0 | 1 |
| EN1 | AMZ2 | 0 | 0 | 1 | 0 | 0 | 0 | 1 |
| FOXJ2 | POU3F4 | 0 | 0 | 0 | 0 | 1 | 0 | 1 |
| FOXJ2 | NECAB3 | 0 | 0 | 0 | 0 | 1 | 0 | 1 |
| EN1 | CDC42EP5 | 0 | 0 | 1 | 0 | 0 | 0 | 1 |
| EN1 | CASC4 | 0 | 0 | 1 | 0 | 0 | 0 | 1 |
| EN1 | TMEM41B | 0 | 0 | 1 | 0 | 0 | 0 | 1 |
| EN1 | UTS2R | 0 | 0 | 1 | 0 | 0 | 0 | 1 |
| EN1 | ASTN2 | 0 | 0 | 1 | 0 | 0 | 0 | 1 |
| EN1 | TMEM30B | 0 | 0 | 1 | 0 | 0 | 0 | 1 |
| FOXJ2 | STOML2 | 0 | 0 | 0 | 0 | 1 | 0 | 1 |
| EN1 | KIAA1644 | 0 | 0 | 1 | 0 | 0 | 0 | 1 |
| EN1 | LAT2 | 0 | 0 | 1 | 0 | 0 | 0 | 1 |
| EN1 | RYBP | 0 | 0 | 1 | 0 | 0 | 0 | 1 |
| FOXJ2 | MEF2C | 0 | 0 | 0 | 0 | 1 | 0 | 1 |
| EN1 | BCL9L | 0 | 0 | 1 | 0 | 0 | 0 | 1 |
| EN1 | KDM4D | 0 | 0 | 0 | 0 | 1 | 0 | 1 |
| FOXJ2 | ARSG | 0 | 0 | 0 | 0 | 1 | 0 | 1 |
| EN1 | ZNF12 | 0 | 0 | 1 | 0 | 0 | 0 | 1 |
| FOXJ2 | ATF3 | 0 | 0 | 0 | 0 | 1 | 0 | 1 |
| EN1 | AARSD1 | 0 | 0 | 1 | 0 | 0 | 0 | 1 |
| EN1 | BTD | 0 | 0 | 1 | 0 | 0 | 0 | 1 |
| EN1 | CLEC3B | 0 | 0 | 1 | 0 | 0 | 0 | 1 |
| EN1 | TTLL2 | 0 | 0 | 1 | 0 | 0 | 0 | 1 |
| EN1 | C6orf15 | 0 | 0 | 1 | 0 | 0 | 0 | 1 |
| EN1 | GJB2 | 0 | 0 | 1 | 0 | 0 | 0 | 1 |
| EN1 | MIR298 | 0 | 0 | 1 | 0 | 0 | 0 | 1 |
| EN1 | KDM6A | 0 | 0 | 0 | 0 | 1 | 0 | 1 |
| FOXJ2 | LEMD2 | 0 | 0 | 0 | 0 | 1 | 0 | 1 |
| EN1 | TMEM64 | 0 | 0 | 1 | 0 | 0 | 0 | 1 |
| FOXJ2 | PVRL3 | 0 | 0 | 0 | 0 | 1 | 0 | 1 |
| EN1 | CLCN3 | 0 | 0 | 1 | 0 | 0 | 0 | 1 |
| EN1 | C1RL | 0 | 0 | 1 | 0 | 0 | 0 | 1 |
| FOXJ2 | DYNC1LI1 | 0 | 0 | 0 | 0 | 1 | 0 | 1 |
| EN1 | CTNNA3 | 0 | 0 | 1 | 0 | 0 | 0 | 1 |
| FOXJ2 | SLC26A7 | 0 | 0 | 0 | 0 | 1 | 0 | 1 |
| FOXJ2 | BRCA2 | 0 | 0 | 0 | 0 | 1 | 0 | 1 |
| EN1 | CYP4F22 | 0 | 0 | 1 | 0 | 0 | 0 | 1 |
| FOXJ2 | FOXB1 | 0 | 0 | 0 | 0 | 1 | 0 | 1 |
| EN1 | HOXD10 | 0 | 0 | 0 | 0 | 1 | 0 | 1 |
| FOXJ2 | ACKR3 | 0 | 0 | 0 | 0 | 1 | 0 | 1 |
| EN1 | HDAC3 | 0 | 0 | 1 | 0 | 0 | 0 | 1 |
| EN1 | MGST2 | 0 | 0 | 1 | 0 | 0 | 0 | 1 |
| EN1 | TINAGL1 | 0 | 0 | 1 | 0 | 0 | 0 | 1 |
| EN1 | RBFOX1 | 0 | 0 | 0 | 0 | 1 | 0 | 1 |
| EN1 | CFAP58 | 0 | 0 | 1 | 0 | 0 | 0 | 1 |
| EN1 | SCRN2 | 0 | 0 | 1 | 0 | 0 | 0 | 1 |
| EN1 | MAP7 | 0 | 0 | 1 | 0 | 0 | 0 | 1 |
| EN1 | EOMES | 0 | 0 | 0 | 0 | 1 | 0 | 1 |
| FOXJ2 | JPH4 | 0 | 0 | 0 | 0 | 1 | 0 | 1 |
| EN1 | EI24 | 0 | 0 | 1 | 0 | 0 | 0 | 1 |
| EN1 | PRSS38 | 0 | 0 | 1 | 0 | 0 | 0 | 1 |
| EN1 | VWA5B2 | 0 | 0 | 1 | 0 | 0 | 0 | 1 |
| FOXJ2 | SCML1 | 0 | 0 | 0 | 0 | 1 | 0 | 1 |
| FOXJ2 | PYGO2 | 0 | 0 | 0 | 0 | 1 | 0 | 1 |
| EN1 | CSTF3 | 0 | 0 | 1 | 0 | 0 | 0 | 1 |
| EN1 | TMCC2 | 0 | 0 | 1 | 0 | 0 | 0 | 1 |
| EN1 | VEGFA | 0 | 0 | 1 | 0 | 0 | 0 | 1 |
| FOXJ2 | ZNF407 | 0 | 0 | 0 | 0 | 1 | 0 | 1 |
| FOXJ2 | EFEMP1 | 0 | 0 | 0 | 0 | 1 | 0 | 1 |
| EN1 | KCNE1 | 0 | 0 | 1 | 0 | 0 | 0 | 1 |
| EN1 | NFYA | 0 | 0 | 1 | 0 | 0 | 0 | 1 |
| EN1 | TEC | 0 | 0 | 1 | 0 | 0 | 0 | 1 |
| FOXJ2 | ZADH2 | 0 | 0 | 0 | 0 | 1 | 0 | 1 |
| EN1 | GPR149 | 0 | 0 | 1 | 0 | 0 | 0 | 1 |
| EN1 | PTCH1 | 0 | 0 | 1 | 0 | 0 | 0 | 1 |
| EN1 | MS4A15 | 0 | 0 | 1 | 0 | 0 | 0 | 1 |
| EN1 | UPK3A | 0 | 0 | 1 | 0 | 0 | 0 | 1 |
| FOXJ2 | TOB1 | 0 | 0 | 0 | 0 | 1 | 0 | 1 |
| EN1 | SPAG11A | 0 | 0 | 1 | 0 | 0 | 0 | 1 |
| EN1 | KRTAP1-5 | 0 | 0 | 1 | 0 | 0 | 0 | 1 |
| EN1 | GJC1 | 0 | 0 | 1 | 0 | 0 | 0 | 1 |
| FOXJ2 | CLDN8 | 0 | 0 | 0 | 0 | 1 | 0 | 1 |
| FOXJ2 | LTA | 0 | 0 | 0 | 0 | 1 | 0 | 1 |
| EN1 | TTC30A | 0 | 0 | 1 | 0 | 0 | 0 | 1 |
| EN1 | ITLN1 | 0 | 0 | 1 | 0 | 0 | 0 | 1 |
| EN1 | MGAT5 | 0 | 0 | 1 | 0 | 0 | 0 | 1 |
| FOXJ2 | CYP26B1 | 0 | 0 | 0 | 0 | 1 | 0 | 1 |
| EN1 | PLEKHG1 | 0 | 0 | 1 | 0 | 0 | 0 | 1 |
| EN1 | SERPINB6 | 0 | 0 | 1 | 0 | 0 | 0 | 1 |
| FOXJ2 | ATXN7L1 | 0 | 0 | 0 | 0 | 1 | 0 | 1 |
| EN1 | MAP3K5 | 0 | 0 | 0 | 0 | 1 | 0 | 1 |
| EN1 | CDK2AP2 | 0 | 0 | 1 | 0 | 0 | 0 | 1 |
| EN1 | IL17RB | 0 | 0 | 1 | 0 | 0 | 0 | 1 |
| EN1 | TRIL | 0 | 0 | 1 | 0 | 0 | 0 | 1 |
| EN1 | KRTAP4-9 | 0 | 0 | 1 | 0 | 0 | 0 | 1 |
| EN1 | LOH12CR1 | 0 | 0 | 1 | 0 | 0 | 0 | 1 |
| EN1 | APOA1 | 0 | 0 | 1 | 0 | 0 | 0 | 1 |
| EN1 | ELN | 0 | 0 | 0 | 0 | 1 | 0 | 1 |
| EN1 | SHCBP1L | 0 | 0 | 1 | 0 | 0 | 0 | 1 |
| EN1 | RASSF3 | 0 | 0 | 1 | 0 | 0 | 0 | 1 |
| EN1 | EPHA8 | 0 | 0 | 1 | 0 | 0 | 0 | 1 |
| FOXJ2 | KRT84 | 0 | 0 | 0 | 0 | 1 | 0 | 1 |
| FOXJ2 | CRIM1 | 0 | 0 | 0 | 0 | 1 | 0 | 1 |
| FOXJ2 | REST | 0 | 0 | 0 | 0 | 1 | 0 | 1 |
| EN1 | NUDT6 | 0 | 0 | 1 | 0 | 0 | 0 | 1 |
| EN1 | FIBCD1 | 0 | 0 | 0 | 0 | 1 | 0 | 1 |
| EN1 | TCTE1 | 0 | 0 | 1 | 0 | 0 | 0 | 1 |
| EN1 | MYOCD | 0 | 0 | 1 | 0 | 0 | 0 | 1 |
| FOXJ2 | PPP1CB | 0 | 0 | 0 | 0 | 1 | 0 | 1 |
| EN1 | NAT8L | 0 | 0 | 1 | 0 | 0 | 0 | 1 |
| EN1 | DNASE2B | 0 | 0 | 1 | 0 | 0 | 0 | 1 |
| EN1 | MIR1912 | 0 | 0 | 1 | 0 | 0 | 0 | 1 |
| FOXJ2 | PPM1D | 0 | 0 | 0 | 0 | 1 | 0 | 1 |
| EN1 | TCN2 | 0 | 0 | 1 | 0 | 0 | 0 | 1 |
| EN1 | IL1RAPL1 | 0 | 0 | 0 | 0 | 1 | 0 | 1 |
| EN1 | TAGAP | 0 | 0 | 1 | 0 | 0 | 0 | 1 |
| FOXJ2 | TCF4 | 0 | 0 | 0 | 0 | 1 | 0 | 1 |
| EN1 | PFDN1 | 0 | 0 | 1 | 0 | 0 | 0 | 1 |
| EN1 | ASNSD1 | 0 | 0 | 1 | 0 | 0 | 0 | 1 |
| EN1 | BCL2L10 | 0 | 0 | 1 | 0 | 0 | 0 | 1 |
| EN1 | CAV2 | 0 | 0 | 1 | 0 | 0 | 0 | 1 |
| EN1 | LRR1 | 0 | 0 | 1 | 0 | 0 | 0 | 1 |
| EN1 | KCNK5 | 0 | 0 | 1 | 0 | 0 | 0 | 1 |
| FOXJ2 | NEO1 | 0 | 0 | 0 | 0 | 1 | 0 | 1 |
| FOXJ2 | MRPL47 | 0 | 0 | 0 | 0 | 1 | 0 | 1 |
| EN1 | RDH10 | 0 | 0 | 0 | 0 | 1 | 0 | 1 |
| EN1 | SERPINI1 | 0 | 0 | 1 | 0 | 0 | 0 | 1 |
| EN1 | ADIPOQ | 0 | 0 | 1 | 0 | 0 | 0 | 1 |
| FOXJ2 | ANGPT1 | 0 | 0 | 0 | 0 | 1 | 0 | 1 |
| EN1 | MIR106A | 0 | 0 | 1 | 0 | 0 | 0 | 1 |
| EN1 | LMO1 | 0 | 0 | 0 | 0 | 1 | 0 | 1 |
| EN1 | CCDC22 | 0 | 0 | 1 | 0 | 0 | 0 | 1 |
| EN1 | ROR2 | 0 | 0 | 1 | 0 | 0 | 0 | 1 |
| EN1 | AFG3L2 | 0 | 0 | 1 | 0 | 0 | 0 | 1 |
| SATB1 | IL5 | 0 | 0 | 0 | 0 | 0 | 1 | 1 |
| EN1 | ANK2 | 0 | 0 | 0 | 0 | 1 | 0 | 1 |
| EN1 | CENPF | 0 | 0 | 1 | 0 | 0 | 0 | 1 |
| EN1 | STK32C | 0 | 0 | 1 | 0 | 0 | 0 | 1 |
| EN1 | ANKRD46 | 0 | 0 | 1 | 0 | 0 | 0 | 1 |
| SATB1 | SPARC | 0 | 0 | 0 | 0 | 0 | 1 | 1 |
| EN1 | PLEKHM2 | 0 | 0 | 1 | 0 | 0 | 0 | 1 |
| EN1 | KCTD14 | 0 | 0 | 1 | 0 | 0 | 0 | 1 |
| EN1 | PPP2R5A | 0 | 0 | 1 | 0 | 0 | 0 | 1 |
| EN1 | MRFAP1 | 0 | 0 | 1 | 0 | 0 | 0 | 1 |
| EN1 | SNORD64 | 0 | 0 | 1 | 0 | 0 | 0 | 1 |
| EN1 | HSBP1L1 | 0 | 0 | 1 | 0 | 0 | 0 | 1 |
| EN1 | ATP9B | 0 | 0 | 0 | 0 | 1 | 0 | 1 |
| EN1 | LRRC29 | 0 | 0 | 1 | 0 | 0 | 0 | 1 |
| EN1 | CLCNKA | 0 | 0 | 1 | 0 | 0 | 0 | 1 |
| EN1 | DHX32 | 0 | 0 | 1 | 0 | 0 | 0 | 1 |
| EN1 | NRAS | 0 | 0 | 0 | 0 | 1 | 0 | 1 |
| FOXJ2 | KIRREL3-AS3 | 0 | 0 | 0 | 0 | 1 | 0 | 1 |
| FOXJ2 | B3GALTL | 0 | 0 | 0 | 0 | 1 | 0 | 1 |
| EN1 | VAMP7 | 0 | 0 | 1 | 0 | 0 | 0 | 1 |
| EN1 | SERHL | 0 | 0 | 1 | 0 | 0 | 0 | 1 |
| FOXJ2 | HEY2 | 0 | 0 | 0 | 0 | 1 | 0 | 1 |
| FOXJ2 | NFIA | 0 | 0 | 0 | 0 | 1 | 0 | 1 |
| EN1 | SCARB1 | 0 | 0 | 1 | 0 | 0 | 0 | 1 |
| EN1 | FAM134C | 0 | 0 | 1 | 0 | 0 | 0 | 1 |
| FOXJ2 | SLC39A14 | 0 | 0 | 0 | 0 | 1 | 0 | 1 |
| EN1 | USPL1 | 0 | 0 | 1 | 0 | 0 | 0 | 1 |
| EN1 | HOXC11 | 0 | 0 | 0 | 0 | 1 | 0 | 1 |
| FOXJ2 | LEMD1 | 0 | 0 | 0 | 0 | 1 | 0 | 1 |
| FOXJ2 | IL20 | 0 | 0 | 0 | 0 | 1 | 0 | 1 |
| FOXJ2 | ADAMTSL1 | 0 | 0 | 0 | 0 | 1 | 0 | 1 |
| EN1 | RAB2B | 0 | 0 | 0 | 0 | 1 | 0 | 1 |
| EN1 | SCAPER | 0 | 0 | 1 | 0 | 0 | 0 | 1 |
| EN1 | MIR337 | 0 | 0 | 1 | 0 | 0 | 0 | 1 |
| FOXJ2 | UGT1A6 | 0 | 0 | 0 | 0 | 1 | 0 | 1 |
| FOXJ2 | ADAM23 | 0 | 0 | 0 | 0 | 1 | 0 | 1 |
| EN1 | PEX11G | 0 | 0 | 1 | 0 | 0 | 0 | 1 |
| EN1 | LAPTM4B | 0 | 0 | 0 | 0 | 1 | 0 | 1 |
| FOXJ2 | IFNA10 | 0 | 0 | 0 | 0 | 1 | 0 | 1 |
| EN1 | RBM4 | 0 | 0 | 0 | 0 | 1 | 0 | 1 |
| EN1 | LGALS3BP | 0 | 0 | 1 | 0 | 0 | 0 | 1 |
| FOXJ2 | INPP4B | 0 | 0 | 0 | 0 | 1 | 0 | 1 |
| EN1 | POLK | 0 | 0 | 1 | 0 | 0 | 0 | 1 |
| EN1 | ST8SIA2 | 0 | 0 | 1 | 0 | 0 | 0 | 1 |
| EN1 | GOLGA7B | 0 | 0 | 1 | 0 | 0 | 0 | 1 |
| EN1 | KIAA2018 | 0 | 0 | 1 | 0 | 0 | 0 | 1 |
| EN1 | PDLIM1 | 0 | 0 | 1 | 0 | 0 | 0 | 1 |
| FOXJ2 | RUNX2 | 0 | 0 | 0 | 0 | 1 | 0 | 1 |
| EN1 | DNAJC28 | 0 | 0 | 1 | 0 | 0 | 0 | 1 |
| EN1 | CREBRF | 0 | 0 | 1 | 0 | 0 | 0 | 1 |
| EN1 | TMEM215 | 0 | 0 | 1 | 0 | 0 | 0 | 1 |
| EN1 | CARS | 0 | 0 | 1 | 0 | 0 | 0 | 1 |
| EN1 | NCALD | 0 | 0 | 1 | 0 | 0 | 0 | 1 |
| FOXJ2 | IFNA5 | 0 | 0 | 0 | 0 | 1 | 0 | 1 |
| EN1 | ARHGAP6 | 0 | 0 | 0 | 0 | 1 | 0 | 1 |
| FOXJ2 | TNR | 0 | 0 | 0 | 0 | 1 | 0 | 1 |
| EN1 | NEDD9 | 0 | 0 | 1 | 0 | 0 | 0 | 1 |
| FOXJ2 | NTN1 | 0 | 0 | 0 | 0 | 1 | 0 | 1 |
| FOXJ2 | CCDC91 | 0 | 0 | 0 | 0 | 1 | 0 | 1 |
| FOXJ2 | COL13A1 | 0 | 0 | 0 | 0 | 1 | 0 | 1 |
| EN1 | SREK1 | 0 | 0 | 0 | 0 | 1 | 0 | 1 |
| EN1 | SPTLC3 | 0 | 0 | 1 | 0 | 0 | 0 | 1 |
| EN1 | C15orf40 | 0 | 0 | 1 | 0 | 0 | 0 | 1 |
| FOXJ2 | ATRNL1 | 0 | 0 | 0 | 0 | 1 | 0 | 1 |
| EN1 | EIF2B3 | 0 | 0 | 1 | 0 | 0 | 0 | 1 |
| EN1 | DONSON | 0 | 0 | 1 | 0 | 0 | 0 | 1 |
| FOXJ2 | GNAZ | 0 | 0 | 0 | 0 | 1 | 0 | 1 |
| FOXJ2 | ICK | 0 | 0 | 0 | 0 | 1 | 0 | 1 |
| EN1 | KIAA1731 | 0 | 0 | 1 | 0 | 0 | 0 | 1 |
| EN1 | EMP1 | 0 | 0 | 1 | 0 | 0 | 0 | 1 |
| EN1 | PAK4 | 0 | 0 | 1 | 0 | 0 | 0 | 1 |
| FOXJ2 | TCF21 | 0 | 0 | 0 | 0 | 1 | 0 | 1 |
| FOXJ2 | PREX2 | 0 | 0 | 0 | 0 | 1 | 0 | 1 |
| EN1 | COPB1 | 0 | 0 | 1 | 0 | 0 | 0 | 1 |
| EN1 | CAMK2B | 0 | 0 | 1 | 0 | 0 | 0 | 1 |
| EN1 | ARFGAP2 | 0 | 0 | 1 | 0 | 0 | 0 | 1 |
| EN1 | KXD1 | 0 | 0 | 1 | 0 | 0 | 0 | 1 |
| EN1 | RBX1 | 0 | 0 | 1 | 0 | 0 | 0 | 1 |
| EN1 | PAPD4 | 0 | 0 | 1 | 0 | 0 | 0 | 1 |
| EN1 | GLOD5 | 0 | 0 | 1 | 0 | 0 | 0 | 1 |
| EN1 | ANKRD36 | 0 | 0 | 1 | 0 | 0 | 0 | 1 |
| FOXJ2 | UBE2E1 | 0 | 0 | 0 | 0 | 1 | 0 | 1 |
| EN1 | DEFB119 | 0 | 0 | 1 | 0 | 0 | 0 | 1 |
| EN1 | MRPS15 | 0 | 0 | 1 | 0 | 0 | 0 | 1 |
| EN1 | NDUFA13 | 0 | 0 | 1 | 0 | 0 | 0 | 1 |
| FOXJ2 | HTR1F | 0 | 0 | 0 | 0 | 1 | 0 | 1 |
| FOXJ2 | RORA | 0 | 0 | 0 | 0 | 1 | 0 | 1 |
| EN1 | BIRC5 | 0 | 0 | 1 | 0 | 0 | 0 | 1 |
| FOXJ2 | PURA | 0 | 0 | 0 | 0 | 1 | 0 | 1 |
| EN1 | CFHR1 | 0 | 0 | 1 | 0 | 0 | 0 | 1 |
| EN1 | C3orf83 | 0 | 0 | 1 | 0 | 0 | 0 | 1 |
| EN1 | MITF | 0 | 0 | 0 | 0 | 1 | 0 | 1 |
| FOXJ2 | SEMA3A | 0 | 0 | 0 | 0 | 1 | 0 | 1 |
| EN1 | ZAK | 0 | 0 | 1 | 0 | 0 | 0 | 1 |
| SATB1 | SPI1 | 0 | 0 | 0 | 0 | 0 | 1 | 1 |
| EN1 | CCK | 0 | 0 | 1 | 0 | 0 | 0 | 1 |
| EN1 | LONP2 | 0 | 0 | 1 | 0 | 0 | 0 | 1 |
| EN1 | UBE2F | 0 | 0 | 1 | 0 | 0 | 0 | 1 |
| FOXJ2 | C12ORF57 | 0 | 0 | 0 | 0 | 1 | 0 | 1 |
| EN1 | FBXO44 | 0 | 0 | 1 | 0 | 0 | 0 | 1 |
| SATB1 | MKI67 | 0 | 0 | 0 | 0 | 0 | 1 | 1 |
| EN1 | LINC00173 | 0 | 0 | 0 | 0 | 1 | 0 | 1 |
| FOXJ2 | FGF9 | 0 | 0 | 0 | 0 | 1 | 0 | 1 |
| FOXJ2 | ORC4 | 0 | 0 | 0 | 0 | 1 | 0 | 1 |
| EN1 | APTX | 0 | 0 | 1 | 0 | 0 | 0 | 1 |
| EN1 | AP2S1 | 0 | 0 | 1 | 0 | 0 | 0 | 1 |
| EN1 | TANK | 0 | 0 | 1 | 0 | 0 | 0 | 1 |
| EN1 | CYP26A1 | 0 | 0 | 0 | 0 | 1 | 0 | 1 |
| EN1 | SOHLH1 | 0 | 0 | 1 | 0 | 0 | 0 | 1 |
| EN1 | NUP107 | 0 | 0 | 1 | 0 | 0 | 0 | 1 |
| FOXJ2 | AMELX | 0 | 0 | 0 | 0 | 1 | 0 | 1 |
| FOXJ2 | IL2RA | 0 | 0 | 0 | 0 | 1 | 0 | 1 |
| EN1 | PRKAG2 | 0 | 0 | 1 | 0 | 0 | 0 | 1 |
| EN1 | UBE2H | 0 | 0 | 0 | 0 | 1 | 0 | 1 |
| FOXJ2 | C2CD2L | 0 | 0 | 0 | 0 | 1 | 0 | 1 |
| EN1 | AIMP2 | 0 | 0 | 1 | 0 | 0 | 0 | 1 |
| EN1 | RGS6 | 0 | 0 | 0 | 0 | 1 | 0 | 1 |
| EN1 | ACSS2 | 0 | 0 | 1 | 0 | 0 | 0 | 1 |
| EN1 | MFAP4 | 0 | 0 | 1 | 0 | 0 | 0 | 1 |
| EN1 | TSHZ2 | 0 | 0 | 1 | 0 | 0 | 0 | 1 |
| EN1 | LIX1L | 0 | 0 | 1 | 0 | 0 | 0 | 1 |
| EN1 | ZIC1 | 0 | 0 | 1 | 0 | 0 | 0 | 1 |
| EN1 | WWP1 | 0 | 0 | 1 | 0 | 0 | 0 | 1 |
| EN1 | IGF2BP2 | 0 | 0 | 1 | 0 | 0 | 0 | 1 |
| FOXJ2 | LIFR | 0 | 0 | 0 | 0 | 1 | 0 | 1 |
| EN1 | GDF2 | 0 | 0 | 1 | 0 | 0 | 0 | 1 |
| FOXJ2 | HPSE2 | 0 | 0 | 0 | 0 | 1 | 0 | 1 |
| EN1 | TUSC5 | 0 | 0 | 1 | 0 | 0 | 0 | 1 |
| FOXJ2 | IGSF21 | 0 | 0 | 0 | 0 | 1 | 0 | 1 |
| EN1 | IQSEC3 | 0 | 0 | 1 | 0 | 0 | 0 | 1 |
| EN1 | TRAM1 | 0 | 0 | 1 | 0 | 0 | 0 | 1 |
| EN1 | UMPS | 0 | 0 | 1 | 0 | 0 | 0 | 1 |
| EN1 | ADAM15 | 0 | 0 | 1 | 0 | 0 | 0 | 1 |
| FOXJ2 | SSH2 | 0 | 0 | 0 | 0 | 1 | 0 | 1 |
| EN1 | NRXN3 | 0 | 0 | 1 | 0 | 0 | 0 | 1 |
| FOXJ2 | MAF | 0 | 0 | 0 | 0 | 1 | 0 | 1 |
| FOXJ2 | CPEB4 | 0 | 0 | 0 | 0 | 1 | 0 | 1 |
| EN1 | PKD1 | 0 | 0 | 1 | 0 | 0 | 0 | 1 |
| FOXJ2 | C1ORF43 | 0 | 0 | 0 | 0 | 1 | 0 | 1 |
| EN1 | C3orf84 | 0 | 0 | 1 | 0 | 0 | 0 | 1 |
| EN1 | ZDHHC21 | 0 | 0 | 1 | 0 | 0 | 0 | 1 |
| FOXJ2 | ZBTB18 | 0 | 0 | 0 | 0 | 1 | 0 | 1 |
| SATB1 | HDAC1 | 0 | 0 | 0 | 0 | 0 | 1 | 1 |
| EN1 | MAPK4 | 0 | 0 | 1 | 0 | 0 | 0 | 1 |
| FOXJ2 | CH25H | 0 | 0 | 0 | 0 | 1 | 0 | 1 |
| FOXJ2 | RAB3IP | 0 | 0 | 0 | 0 | 1 | 0 | 1 |
| EN1 | PAG1 | 0 | 0 | 1 | 0 | 0 | 0 | 1 |
| EN1 | MAFB | 0 | 0 | 1 | 0 | 0 | 0 | 1 |
| FOXJ2 | SLN | 0 | 0 | 0 | 0 | 1 | 0 | 1 |
| FOXJ2 | RPS6KB1 | 0 | 0 | 0 | 0 | 1 | 0 | 1 |
| EN1 | MOXD1 | 0 | 0 | 1 | 0 | 0 | 0 | 1 |
| EN1 | SPINT1 | 0 | 0 | 1 | 0 | 0 | 0 | 1 |
| EN1 | SYNCRIP | 0 | 0 | 1 | 0 | 0 | 0 | 1 |
| EN1 | PCDHB11 | 0 | 0 | 1 | 0 | 0 | 0 | 1 |
| EN1 | AQPEP | 0 | 0 | 1 | 0 | 0 | 0 | 1 |
| EN1 | CHRM4 | 0 | 0 | 1 | 0 | 0 | 0 | 1 |
| EN1 | OR2C1 | 0 | 0 | 1 | 0 | 0 | 0 | 1 |
| EN1 | MAGEA3 | 0 | 0 | 1 | 0 | 0 | 0 | 1 |
| EN1 | OPRD1 | 0 | 0 | 1 | 0 | 0 | 0 | 1 |
| EN1 | RIPK4 | 0 | 0 | 1 | 0 | 0 | 0 | 1 |
| EN1 | PIK3AP1 | 0 | 0 | 1 | 0 | 0 | 0 | 1 |
| EN1 | FLT4 | 0 | 0 | 1 | 0 | 0 | 0 | 1 |
| FOXJ2 | GAP43 | 0 | 0 | 0 | 0 | 1 | 0 | 1 |
| FOXJ2 | SPTLC2 | 0 | 0 | 0 | 0 | 1 | 0 | 1 |
| EN1 | SSSCA1 | 0 | 0 | 1 | 0 | 0 | 0 | 1 |
| EN1 | SERPINB13 | 0 | 0 | 1 | 0 | 0 | 0 | 1 |
| EN1 | RGS22 | 0 | 0 | 1 | 0 | 0 | 0 | 1 |
| FOXJ2 | SATB2 | 0 | 0 | 0 | 0 | 1 | 0 | 1 |
| FOXJ2 | LCP2 | 0 | 0 | 0 | 0 | 1 | 0 | 1 |
| FOXJ2 | IL17C | 0 | 0 | 0 | 0 | 1 | 0 | 1 |
| EN1 | FNDC3A | 0 | 0 | 0 | 0 | 1 | 0 | 1 |
| FOXJ2 | CTNNAL1 | 0 | 0 | 0 | 0 | 1 | 0 | 1 |
| EN1 | SERPINB3 | 0 | 0 | 1 | 0 | 0 | 0 | 1 |
| EN1 | HS1BP3 | 0 | 0 | 1 | 0 | 0 | 0 | 1 |
| EN1 | MAPK14 | 0 | 0 | 0 | 0 | 1 | 0 | 1 |
| EN1 | CABIN1 | 0 | 0 | 1 | 0 | 0 | 0 | 1 |
| EN1 | KRT222 | 0 | 0 | 1 | 0 | 0 | 0 | 1 |
| FOXJ2 | PHTF2 | 0 | 0 | 0 | 0 | 1 | 0 | 1 |
| EN1 | RUNX1T1 | 0 | 0 | 0 | 0 | 1 | 0 | 1 |
| EN1 | RPH3AL | 0 | 0 | 1 | 0 | 0 | 0 | 1 |
| FOXJ2 | AAMP | 0 | 0 | 0 | 0 | 1 | 0 | 1 |
| EN1 | NFKB2 | 0 | 0 | 1 | 0 | 0 | 0 | 1 |
| EN1 | NOTCH1 | 0 | 0 | 0 | 0 | 1 | 0 | 1 |
| EN1 | MAP7D3 | 0 | 0 | 1 | 0 | 0 | 0 | 1 |
| EN1 | TTC13 | 0 | 0 | 1 | 0 | 0 | 0 | 1 |
| EN1 | DAPL1 | 0 | 0 | 1 | 0 | 0 | 0 | 1 |
| EN1 | CD276 | 0 | 0 | 1 | 0 | 0 | 0 | 1 |
| FOXJ2 | CPNE1 | 0 | 0 | 0 | 0 | 1 | 0 | 1 |
| EN1 | MXI1 | 0 | 0 | 1 | 0 | 0 | 0 | 1 |
| EN1 | LRFN4 | 0 | 0 | 1 | 0 | 0 | 0 | 1 |
| EN1 | TEAD1 | 0 | 0 | 1 | 0 | 0 | 0 | 1 |
| EN1 | GLIS1 | 0 | 0 | 1 | 0 | 0 | 0 | 1 |
| EN1 | OR5L1 | 0 | 0 | 1 | 0 | 0 | 0 | 1 |
| FOXJ2 | FAM72A | 0 | 0 | 0 | 0 | 1 | 0 | 1 |
| FOXJ2 | TFAP2B | 0 | 0 | 0 | 0 | 1 | 0 | 1 |
| FOXJ2 | SAR1B | 0 | 0 | 0 | 0 | 1 | 0 | 1 |
| EN1 | FAM78B | 0 | 0 | 1 | 0 | 0 | 0 | 1 |
| EN1 | ADCY4 | 0 | 0 | 1 | 0 | 0 | 0 | 1 |
| EN1 | PRKCDBP | 0 | 0 | 1 | 0 | 0 | 0 | 1 |
| EN1 | NLGN2 | 0 | 0 | 1 | 0 | 0 | 0 | 1 |
| EN1 | MLC1 | 0 | 0 | 1 | 0 | 0 | 0 | 1 |
| FOXJ2 | C6ORF136 | 0 | 0 | 0 | 0 | 1 | 0 | 1 |
| FOXJ2 | FOXP1 | 0 | 0 | 0 | 0 | 1 | 0 | 1 |
| EN1 | SCHIP1 | 0 | 0 | 1 | 0 | 0 | 0 | 1 |
| EN1 | PLCD4 | 0 | 0 | 1 | 0 | 0 | 0 | 1 |
| EN1 | PDIK1L | 0 | 0 | 1 | 0 | 0 | 0 | 1 |
| EN1 | FOXC1 | 0 | 0 | 1 | 0 | 0 | 0 | 1 |
| EN1 | FBLL1 | 0 | 0 | 1 | 0 | 0 | 0 | 1 |
| EN1 | TYMS | 0 | 0 | 1 | 0 | 0 | 0 | 1 |
| EN1 | NUDT16 | 0 | 0 | 1 | 0 | 0 | 0 | 1 |
| EN1 | SRF | 0 | 0 | 1 | 0 | 0 | 0 | 1 |
| EN1 | GPR183 | 0 | 0 | 1 | 0 | 0 | 0 | 1 |
| EN1 | C1orf74 | 0 | 0 | 1 | 0 | 0 | 0 | 1 |
| SATB1 | CDK4 | 0 | 0 | 0 | 0 | 0 | 1 | 1 |
| EN1 | SNX8 | 0 | 0 | 1 | 0 | 0 | 0 | 1 |
| EN1 | NUDT21 | 0 | 0 | 1 | 0 | 0 | 0 | 1 |
| EN1 | PATE3 | 0 | 0 | 1 | 0 | 0 | 0 | 1 |
| EN1 | PARD6G | 0 | 0 | 1 | 0 | 0 | 0 | 1 |
| EN1 | SIAH3 | 0 | 0 | 1 | 0 | 0 | 0 | 1 |
| FOXJ2 | SLC39A8 | 0 | 0 | 0 | 0 | 1 | 0 | 1 |
| EN1 | CWC15 | 0 | 0 | 0 | 0 | 1 | 0 | 1 |
| FOXJ2 | PVRL1 | 0 | 0 | 0 | 0 | 1 | 0 | 1 |
| EN1 | RAD17 | 0 | 0 | 1 | 0 | 0 | 0 | 1 |
| EN1 | NPDC1 | 0 | 0 | 1 | 0 | 0 | 0 | 1 |
| FOXJ2 | DNAJB12 | 0 | 0 | 0 | 0 | 1 | 0 | 1 |
| EN1 | F8A1 | 0 | 0 | 1 | 0 | 0 | 0 | 1 |
| EN1 | PSME4 | 0 | 0 | 1 | 0 | 0 | 0 | 1 |
| SATB1 | IL2 | 0 | 0 | 0 | 0 | 0 | 1 | 1 |
| EN1 | RTKN | 0 | 0 | 0 | 0 | 1 | 0 | 1 |
| EN1 | DOK4 | 0 | 0 | 1 | 0 | 0 | 0 | 1 |
| EN1 | SLC34A1 | 0 | 0 | 1 | 0 | 0 | 0 | 1 |
| EN1 | KLHDC9 | 0 | 0 | 1 | 0 | 0 | 0 | 1 |
| EN1 | PCBP3 | 0 | 0 | 1 | 0 | 0 | 0 | 1 |
| FOXJ2 | PBX1 | 0 | 0 | 0 | 0 | 1 | 0 | 1 |
| EN1 | RSRC2 | 0 | 0 | 1 | 0 | 0 | 0 | 1 |
| EN1 | PEX5L | 0 | 0 | 1 | 0 | 0 | 0 | 1 |
| EN1 | S100A2 | 0 | 0 | 1 | 0 | 0 | 0 | 1 |
| FOXJ2 | DOCK3 | 0 | 0 | 0 | 0 | 1 | 0 | 1 |
| EN1 | RARB | 0 | 0 | 0 | 0 | 1 | 0 | 1 |
| FOXJ2 | KLHL1 | 0 | 0 | 0 | 0 | 1 | 0 | 1 |
| FOXJ2 | EDN1 | 0 | 0 | 0 | 0 | 1 | 0 | 1 |
| EN1 | ARCN1 | 0 | 0 | 1 | 0 | 0 | 0 | 1 |
| FOXJ2 | DCDC5 | 0 | 0 | 0 | 0 | 1 | 0 | 1 |
| EN1 | GABRB3 | 0 | 0 | 1 | 0 | 0 | 0 | 1 |
| EN1 | ATP2B2 | 0 | 0 | 1 | 0 | 0 | 0 | 1 |
| EN1 | NOS1AP | 0 | 0 | 1 | 0 | 0 | 0 | 1 |
| EN1 | FAM160B1 | 0 | 0 | 1 | 0 | 0 | 0 | 1 |
| EN1 | MRPL38 | 0 | 0 | 1 | 0 | 0 | 0 | 1 |
| EN1 | PCDH9 | 0 | 0 | 0 | 0 | 1 | 0 | 1 |
| FOXJ2 | PDE7A | 0 | 0 | 0 | 0 | 1 | 0 | 1 |
| EN1 | RELB | 0 | 0 | 1 | 0 | 0 | 0 | 1 |
| FOXJ2 | PSMA8 | 0 | 0 | 0 | 0 | 1 | 0 | 1 |
| EN1 | NKX2-2 | 0 | 0 | 0 | 0 | 1 | 0 | 1 |
| EN1 | AP4M1 | 0 | 0 | 1 | 0 | 0 | 0 | 1 |
| EN1 | F2RL3 | 0 | 0 | 1 | 0 | 0 | 0 | 1 |
| EN1 | MMP14 | 0 | 0 | 0 | 0 | 1 | 0 | 1 |
| EN1 | SRM | 0 | 0 | 1 | 0 | 0 | 0 | 1 |
| FOXJ2 | NRXN1 | 0 | 0 | 0 | 0 | 1 | 0 | 1 |
| EN1 | SLC6A14 | 0 | 0 | 0 | 0 | 1 | 0 | 1 |
| FOXJ2 | GFRA1 | 0 | 0 | 0 | 0 | 1 | 0 | 1 |
| EN1 | KRTAP9-3 | 0 | 0 | 1 | 0 | 0 | 0 | 1 |
| EN1 | ATP6V0A2 | 0 | 0 | 1 | 0 | 0 | 0 | 1 |
| EN1 | GJA1 | 0 | 0 | 0 | 0 | 1 | 0 | 1 |
| EN1 | ZFP82 | 0 | 0 | 1 | 0 | 0 | 0 | 1 |
| SLC2A4RG | SLC2A4 | 0 | 0 | 0 | 0 | 0 | 1 | 1 |
| EN1 | CARD10 | 0 | 0 | 1 | 0 | 0 | 0 | 1 |
| FOXJ2 | TFEC | 0 | 0 | 0 | 0 | 1 | 0 | 1 |
| EN1 | POLR3B | 0 | 0 | 1 | 0 | 0 | 0 | 1 |
| EN1 | NUP54 | 0 | 0 | 1 | 0 | 0 | 0 | 1 |
| EN1 | CASR | 0 | 0 | 1 | 0 | 0 | 0 | 1 |
| EN1 | ZC3H12D | 0 | 0 | 1 | 0 | 0 | 0 | 1 |
| FOXJ2 | KLF12 | 0 | 0 | 0 | 0 | 1 | 0 | 1 |
| EN1 | PLEKHA2 | 0 | 0 | 1 | 0 | 0 | 0 | 1 |
| FOXJ2 | DNAJB9 | 0 | 0 | 0 | 0 | 1 | 0 | 1 |
| EN1 | SLC17A9 | 0 | 0 | 1 | 0 | 0 | 0 | 1 |
| EN1 | OR5AN1 | 0 | 0 | 1 | 0 | 0 | 0 | 1 |
| EN1 | SCARNA17 | 0 | 0 | 1 | 0 | 0 | 0 | 1 |
| EN1 | GRIA4 | 0 | 0 | 1 | 0 | 0 | 0 | 1 |
| FOXJ2 | LMO3 | 0 | 0 | 0 | 0 | 1 | 0 | 1 |
| EN1 | TYMP | 0 | 0 | 1 | 0 | 0 | 0 | 1 |
| EN1 | PPP4R1 | 0 | 0 | 1 | 0 | 0 | 0 | 1 |
| EN1 | SMAD5 | 0 | 0 | 1 | 0 | 0 | 0 | 1 |
| EN1 | HOXD3 | 0 | 0 | 0 | 0 | 1 | 0 | 1 |
| FOXJ2 | CCL2 | 0 | 0 | 0 | 0 | 1 | 0 | 1 |
| EN1 | CCND2 | 0 | 0 | 1 | 0 | 0 | 0 | 1 |
| EN1 | SIRPA | 0 | 0 | 1 | 0 | 0 | 0 | 1 |
| EN1 | TEP1 | 0 | 0 | 1 | 0 | 0 | 0 | 1 |
| EN1 | PLEKHA4 | 0 | 0 | 1 | 0 | 0 | 0 | 1 |
| EN1 | RNF144A | 0 | 0 | 1 | 0 | 0 | 0 | 1 |
| EN1 | PLEKHA8 | 0 | 0 | 1 | 0 | 0 | 0 | 1 |
| EN1 | TAF2 | 0 | 0 | 1 | 0 | 0 | 0 | 1 |
| EN1 | TMPRSS3 | 0 | 0 | 1 | 0 | 0 | 0 | 1 |
| FOXJ2 | NOG | 0 | 0 | 0 | 0 | 1 | 0 | 1 |
| FOXJ2 | ATP2A3 | 0 | 0 | 0 | 0 | 1 | 0 | 1 |
| FOXJ2 | OTX2 | 0 | 0 | 0 | 0 | 1 | 0 | 1 |
| EN1 | TSC22D1 | 0 | 0 | 1 | 0 | 0 | 0 | 1 |
| EN1 | FOXK2 | 0 | 0 | 1 | 0 | 0 | 0 | 1 |
| EN1 | BTNL2 | 0 | 0 | 1 | 0 | 0 | 0 | 1 |
| FOXJ2 | DMD | 0 | 0 | 0 | 0 | 1 | 0 | 1 |
| EN1 | HEY2 | 0 | 0 | 1 | 0 | 0 | 0 | 1 |
| EN1 | HECW1 | 0 | 0 | 1 | 0 | 0 | 0 | 1 |
| FOXJ2 | RGMA | 0 | 0 | 0 | 0 | 1 | 0 | 1 |
| EN1 | ELTD1 | 0 | 0 | 1 | 0 | 0 | 0 | 1 |
| FOXJ2 | BDH1 | 0 | 0 | 0 | 0 | 1 | 0 | 1 |
| FOXJ2 | NOL4L | 0 | 0 | 0 | 0 | 1 | 0 | 1 |
| EN1 | EIF3M | 0 | 0 | 1 | 0 | 0 | 0 | 1 |
| FOXJ2 | ACP6 | 0 | 0 | 0 | 0 | 1 | 0 | 1 |
| EN1 | EFEMP1 | 0 | 0 | 0 | 0 | 1 | 0 | 1 |
| EN1 | LRRC23 | 0 | 0 | 1 | 0 | 0 | 0 | 1 |
| EN1 | SCAMP5 | 0 | 0 | 1 | 0 | 0 | 0 | 1 |
| FOXJ2 | HIATL1 | 0 | 0 | 0 | 0 | 1 | 0 | 1 |
| EN1 | FBXL21 | 0 | 0 | 1 | 0 | 0 | 0 | 1 |
| FOXJ2 | UCHL3 | 0 | 0 | 0 | 0 | 1 | 0 | 1 |
| EN1 | SBSPON | 0 | 0 | 1 | 0 | 0 | 0 | 1 |
| EN1 | MORC4 | 0 | 0 | 1 | 0 | 0 | 0 | 1 |
| FOXJ2 | TRIB2 | 0 | 0 | 0 | 0 | 1 | 0 | 1 |
| EN1 | ANKZF1 | 0 | 0 | 1 | 0 | 0 | 0 | 1 |
| FOXJ2 | NTF3 | 0 | 0 | 0 | 0 | 1 | 0 | 1 |
| FOXJ2 | SLC44A1 | 0 | 0 | 0 | 0 | 1 | 0 | 1 |
| EN1 | PDZK1 | 0 | 0 | 1 | 0 | 0 | 0 | 1 |
| EN1 | ANXA13 | 0 | 0 | 1 | 0 | 0 | 0 | 1 |
| EN1 | EPB41L2 | 0 | 0 | 1 | 0 | 0 | 0 | 1 |
| EN1 | B4GALT2 | 0 | 0 | 1 | 0 | 0 | 0 | 1 |
| EN1 | ITPRIPL2 | 0 | 0 | 1 | 0 | 0 | 0 | 1 |
| EN1 | PARVB | 0 | 0 | 1 | 0 | 0 | 0 | 1 |
| EN1 | XIAP | 0 | 0 | 1 | 0 | 0 | 0 | 1 |
| EN1 | HMGB3 | 0 | 0 | 1 | 0 | 0 | 0 | 1 |
| FOXJ2 | ATP2B4 | 0 | 0 | 0 | 0 | 1 | 0 | 1 |
| FOXJ2 | IER3 | 0 | 0 | 0 | 0 | 1 | 0 | 1 |
| EN1 | SLCO1C1 | 0 | 0 | 1 | 0 | 0 | 0 | 1 |
| EN1 | LRRC32 | 0 | 0 | 1 | 0 | 0 | 0 | 1 |
| EN1 | ADAMTSL5 | 0 | 0 | 1 | 0 | 0 | 0 | 1 |
| EN1 | ACVR1C | 0 | 0 | 1 | 0 | 0 | 0 | 1 |
| EN1 | HDHD3 | 0 | 0 | 1 | 0 | 0 | 0 | 1 |
| EN1 | IRX4 | 0 | 0 | 0 | 0 | 1 | 0 | 1 |
| FOXJ2 | TSPYL2 | 0 | 0 | 0 | 0 | 1 | 0 | 1 |
| EN1 | LMBRD2 | 0 | 0 | 1 | 0 | 0 | 0 | 1 |
| EN1 | SFI1 | 0 | 0 | 1 | 0 | 0 | 0 | 1 |
| EN1 | JAKMIP2 | 0 | 0 | 0 | 0 | 1 | 0 | 1 |
| FOXJ2 | CNTLN | 0 | 0 | 0 | 0 | 1 | 0 | 1 |
| FOXJ2 | TTC3 | 0 | 0 | 0 | 0 | 1 | 0 | 1 |
| EN1 | RASSF9 | 0 | 0 | 0 | 0 | 1 | 0 | 1 |
| EN1 | SSPO | 0 | 0 | 1 | 0 | 0 | 0 | 1 |
| FOXJ2 | TBX5 | 0 | 0 | 0 | 0 | 1 | 0 | 1 |
| FOXJ2 | IGFBP7 | 0 | 0 | 0 | 0 | 1 | 0 | 1 |
| FOXJ2 | SCAMP1 | 0 | 0 | 0 | 0 | 1 | 0 | 1 |
| EN1 | DCTD | 0 | 0 | 1 | 0 | 0 | 0 | 1 |
| EN1 | CCL4 | 0 | 0 | 1 | 0 | 0 | 0 | 1 |
| FOXJ2 | BRIP1 | 0 | 0 | 0 | 0 | 1 | 0 | 1 |
| EN1 | CYTL1 | 0 | 0 | 1 | 0 | 0 | 0 | 1 |
| FOXJ2 | CHD2 | 0 | 0 | 0 | 0 | 1 | 0 | 1 |
| FOXJ2 | DLX2 | 0 | 0 | 0 | 0 | 1 | 0 | 1 |
| EN1 | CD151 | 0 | 0 | 1 | 0 | 0 | 0 | 1 |
| EN1 | TMEM56 | 0 | 0 | 1 | 0 | 0 | 0 | 1 |
| EN1 | ATF3 | 0 | 0 | 1 | 0 | 0 | 0 | 1 |
| FOXJ2 | ATP1B4 | 0 | 0 | 0 | 0 | 1 | 0 | 1 |
| EN1 | OSTC | 0 | 0 | 1 | 0 | 0 | 0 | 1 |
| FOXJ2 | CDK14 | 0 | 0 | 0 | 0 | 1 | 0 | 1 |
| EN1 | HELT | 0 | 0 | 1 | 0 | 0 | 0 | 1 |
| EN1 | CSF1R | 0 | 0 | 1 | 0 | 0 | 0 | 1 |
| FOXJ2 | GPM6B | 0 | 0 | 0 | 0 | 1 | 0 | 1 |
| EN1 | GAS6 | 0 | 0 | 1 | 0 | 0 | 0 | 1 |
| EN1 | FHL1 | 0 | 0 | 0 | 0 | 1 | 0 | 1 |
| FOXJ2 | KIRREL3 | 0 | 0 | 0 | 0 | 1 | 0 | 1 |
| FOXJ2 | NKX2-1 | 0 | 0 | 0 | 0 | 1 | 0 | 1 |
| FOXJ2 | GUCY2C | 0 | 0 | 0 | 0 | 1 | 0 | 1 |
| EN1 | PEX10 | 0 | 0 | 1 | 0 | 0 | 0 | 1 |
| FOXJ2 | DTNA | 0 | 0 | 0 | 0 | 1 | 0 | 1 |
| EN1 | CAP2 | 0 | 0 | 1 | 0 | 0 | 0 | 1 |
| EN1 | RPS15A | 0 | 0 | 1 | 0 | 0 | 0 | 1 |
| FOXJ2 | DND1 | 0 | 0 | 0 | 0 | 1 | 0 | 1 |
| FOXJ2 | PTPRO | 0 | 0 | 0 | 0 | 1 | 0 | 1 |
| EN1 | GBP1 | 0 | 0 | 1 | 0 | 0 | 0 | 1 |
| FOXJ2 | SLC10A7 | 0 | 0 | 0 | 0 | 1 | 0 | 1 |
| FOXJ2 | MLH3 | 0 | 0 | 0 | 0 | 1 | 0 | 1 |
| EN1 | P2RY6 | 0 | 0 | 1 | 0 | 0 | 0 | 1 |
| FOXJ2 | RGS6 | 0 | 0 | 0 | 0 | 1 | 0 | 1 |
| FOXJ2 | E2F5 | 0 | 0 | 0 | 0 | 1 | 0 | 1 |
| EN1 | C17orf66 | 0 | 0 | 1 | 0 | 0 | 0 | 1 |
| EN1 | GALK1 | 0 | 0 | 1 | 0 | 0 | 0 | 1 |
| FOXJ2 | ID2 | 0 | 0 | 0 | 0 | 1 | 0 | 1 |
| EN1 | CTNNA2 | 0 | 0 | 1 | 0 | 0 | 0 | 1 |
| FOXJ2 | ATF7 | 0 | 0 | 0 | 0 | 1 | 0 | 1 |
| EN1 | SLC1A1 | 0 | 0 | 1 | 0 | 0 | 0 | 1 |
| FOXJ2 | PTF1A | 0 | 0 | 0 | 0 | 1 | 0 | 1 |
| EN1 | CD55 | 0 | 0 | 0 | 0 | 1 | 0 | 1 |
| EN1 | GRIA3 | 0 | 0 | 1 | 0 | 0 | 0 | 1 |
| FOXJ2 | FCHSD2 | 0 | 0 | 0 | 0 | 1 | 0 | 1 |
| EN1 | RPS6KA1 | 0 | 0 | 1 | 0 | 0 | 0 | 1 |
| EN1 | HARBI1 | 0 | 0 | 1 | 0 | 0 | 0 | 1 |
| FOXJ2 | FBXO11 | 0 | 0 | 0 | 0 | 1 | 0 | 1 |
| EN1 | ADARB1 | 0 | 0 | 1 | 0 | 0 | 0 | 1 |
| FOXJ2 | FAM122A | 0 | 0 | 0 | 0 | 1 | 0 | 1 |
| EN1 | HSD3B2 | 0 | 0 | 1 | 0 | 0 | 0 | 1 |
| FOXJ2 | NOVA1 | 0 | 0 | 0 | 0 | 1 | 0 | 1 |
| FOXJ2 | ZFHX4 | 0 | 0 | 0 | 0 | 1 | 0 | 1 |
| EN1 | DR1 | 0 | 0 | 1 | 0 | 0 | 0 | 1 |
| EN1 | G6PC2 | 0 | 0 | 1 | 0 | 0 | 0 | 1 |
| EN1 | TRIOBP | 0 | 0 | 1 | 0 | 0 | 0 | 1 |
| EN1 | TCAIM | 0 | 0 | 1 | 0 | 0 | 0 | 1 |
| EN1 | FBXL3 | 0 | 0 | 1 | 0 | 0 | 0 | 1 |
| EN1 | OOEP | 0 | 0 | 1 | 0 | 0 | 0 | 1 |
| EN1 | TGM1 | 0 | 0 | 1 | 0 | 0 | 0 | 1 |
| FOXJ2 | KIAA0355 | 0 | 0 | 0 | 0 | 1 | 0 | 1 |
| EN1 | HOXA11 | 0 | 0 | 0 | 0 | 1 | 0 | 1 |
| EN1 | WNT8A | 0 | 0 | 1 | 0 | 0 | 0 | 1 |
| EN1 | EDRF1 | 0 | 0 | 1 | 0 | 0 | 0 | 1 |
| EN1 | OTX2 | 0 | 0 | 0 | 0 | 1 | 0 | 1 |
| EN1 | GALR1 | 0 | 0 | 1 | 0 | 0 | 0 | 1 |
| FOXJ2 | GOLGA1 | 0 | 0 | 0 | 0 | 1 | 0 | 1 |
| FOXJ2 | BMI1 | 0 | 0 | 0 | 0 | 1 | 0 | 1 |
| EN1 | ABHD17A | 0 | 0 | 1 | 0 | 0 | 0 | 1 |
| EN1 | AFF4 | 0 | 0 | 0 | 0 | 1 | 0 | 1 |
| EN1 | KLF4 | 0 | 0 | 1 | 0 | 0 | 0 | 1 |
| EN1 | CYP4V2 | 0 | 0 | 1 | 0 | 0 | 0 | 1 |
| EN1 | TMEM14C | 0 | 0 | 1 | 0 | 0 | 0 | 1 |
| EN1 | MCM3 | 0 | 0 | 1 | 0 | 0 | 0 | 1 |
| FOXJ2 | ANGPTL1 | 0 | 0 | 0 | 0 | 1 | 0 | 1 |
| EN1 | SUGP1 | 0 | 0 | 1 | 0 | 0 | 0 | 1 |
| EN1 | ELAVL4 | 0 | 0 | 1 | 0 | 0 | 0 | 1 |
| EN1 | KRTAP4-3 | 0 | 0 | 1 | 0 | 0 | 0 | 1 |
| EN1 | MB | 0 | 0 | 1 | 0 | 0 | 0 | 1 |
| EN1 | UTRN | 0 | 0 | 0 | 0 | 0 | 1 | 1 |
| FOXJ2 | SOX2 | 0 | 0 | 0 | 0 | 1 | 0 | 1 |
| EN1 | CDC73 | 0 | 0 | 1 | 0 | 0 | 0 | 1 |
| EN1 | ZNF274 | 0 | 0 | 1 | 0 | 0 | 0 | 1 |
| EN1 | MEIS1 | 0 | 0 | 1 | 0 | 0 | 0 | 1 |
| FOXJ2 | BUB1 | 0 | 0 | 0 | 0 | 1 | 0 | 1 |
| FOXJ2 | TRERF1 | 0 | 0 | 0 | 0 | 1 | 0 | 1 |
| EN1 | ZCCHC7 | 0 | 0 | 1 | 0 | 0 | 0 | 1 |
| FOXJ2 | HOXA7 | 0 | 0 | 0 | 0 | 1 | 0 | 1 |
| EN1 | COX5A | 0 | 0 | 1 | 0 | 0 | 0 | 1 |
| EN1 | CALN1 | 0 | 0 | 1 | 0 | 0 | 0 | 1 |
| EN1 | MIXL1 | 0 | 0 | 1 | 0 | 0 | 0 | 1 |
| EN1 | HLA-DQA1 | 0 | 0 | 1 | 0 | 0 | 0 | 1 |
| FOXJ2 | CREB5 | 0 | 0 | 0 | 0 | 1 | 0 | 1 |
| FOXJ2 | HOXA9 | 0 | 0 | 0 | 0 | 1 | 0 | 1 |
| FOXJ2 | TGFB2 | 0 | 0 | 0 | 0 | 1 | 0 | 1 |
| EN1 | DCUN1D3 | 0 | 0 | 1 | 0 | 0 | 0 | 1 |
| FOXJ2 | PTCHD4 | 0 | 0 | 0 | 0 | 1 | 0 | 1 |
| FOXJ2 | SLC35C2 | 0 | 0 | 0 | 0 | 1 | 0 | 1 |
| FOXJ2 | MSTN | 0 | 0 | 0 | 0 | 1 | 0 | 1 |
| EN1 | ENTPD4 | 0 | 0 | 1 | 0 | 0 | 0 | 1 |
| EN1 | RNF39 | 0 | 0 | 0 | 0 | 1 | 0 | 1 |
| EN1 | C9orf169 | 0 | 0 | 1 | 0 | 0 | 0 | 1 |
| EN1 | SMPX | 0 | 0 | 1 | 0 | 0 | 0 | 1 |
| EN1 | SPAG17 | 0 | 0 | 1 | 0 | 0 | 0 | 1 |
| EN1 | CDC14A | 0 | 0 | 0 | 0 | 1 | 0 | 1 |
| EN1 | SLC9A3R1 | 0 | 0 | 1 | 0 | 0 | 0 | 1 |
| EN1 | CCL28 | 0 | 0 | 1 | 0 | 0 | 0 | 1 |
| EN1 | AGER | 0 | 0 | 1 | 0 | 0 | 0 | 1 |
| FOXJ2 | TXLNG | 0 | 0 | 0 | 0 | 1 | 0 | 1 |
| EN1 | CLDND1 | 0 | 0 | 1 | 0 | 0 | 0 | 1 |
| EN1 | CKAP2L | 0 | 0 | 1 | 0 | 0 | 0 | 1 |
| EN1 | RERG | 0 | 0 | 1 | 0 | 0 | 0 | 1 |
| FOXJ2 | PDZD2 | 0 | 0 | 0 | 0 | 1 | 0 | 1 |
| FOXJ2 | KCTD15 | 0 | 0 | 0 | 0 | 1 | 0 | 1 |
| FOXJ2 | PAX6 | 0 | 0 | 0 | 0 | 1 | 0 | 1 |
| EN1 | CHN1 | 0 | 0 | 1 | 0 | 0 | 0 | 1 |
| EN1 | KIF2A | 0 | 0 | 1 | 0 | 0 | 0 | 1 |
| EN1 | REXO2 | 0 | 0 | 1 | 0 | 0 | 0 | 1 |
| FOXJ2 | PDE4B | 0 | 0 | 0 | 0 | 1 | 0 | 1 |
| EN1 | ZNF622 | 0 | 0 | 1 | 0 | 0 | 0 | 1 |
| EN1 | IL18R1 | 0 | 0 | 1 | 0 | 0 | 0 | 1 |
| EN1 | OPN4 | 0 | 0 | 1 | 0 | 0 | 0 | 1 |
| FOXJ2 | PHF6 | 0 | 0 | 0 | 0 | 1 | 0 | 1 |
| EN1 | THBS1 | 0 | 0 | 1 | 0 | 0 | 0 | 1 |
| EN1 | VRTN | 0 | 0 | 1 | 0 | 0 | 0 | 1 |
| EN1 | CST3 | 0 | 0 | 1 | 0 | 0 | 0 | 1 |
| EN1 | LHX5 | 0 | 0 | 1 | 0 | 0 | 0 | 1 |
| FOXJ2 | PCYT1B | 0 | 0 | 0 | 0 | 1 | 0 | 1 |
| EN1 | NRM | 0 | 0 | 1 | 0 | 0 | 0 | 1 |
| FOXJ2 | NRG1 | 0 | 0 | 0 | 0 | 1 | 0 | 1 |
| EN1 | ERBB4 | 0 | 0 | 0 | 0 | 1 | 0 | 1 |
| EN1 | RNH1 | 0 | 0 | 1 | 0 | 0 | 0 | 1 |
| EN1 | CDH19 | 0 | 0 | 1 | 0 | 0 | 0 | 1 |
| EN1 | ACO2 | 0 | 0 | 1 | 0 | 0 | 0 | 1 |
| FOXJ2 | TLE4 | 0 | 0 | 0 | 0 | 1 | 0 | 1 |
| EN1 | PLCB1 | 0 | 0 | 0 | 0 | 1 | 0 | 1 |
| EN1 | PDGFRA | 0 | 0 | 0 | 0 | 1 | 0 | 1 |
| FOXJ2 | LRP1B | 0 | 0 | 0 | 0 | 1 | 0 | 1 |
| FOXJ2 | CRYGB | 0 | 0 | 0 | 0 | 1 | 0 | 1 |
| EN1 | MIR381 | 0 | 0 | 1 | 0 | 0 | 0 | 1 |
| EN1 | DLEU7 | 0 | 0 | 1 | 0 | 0 | 0 | 1 |
| EN1 | HTR7 | 0 | 0 | 0 | 0 | 1 | 0 | 1 |
| FOXJ2 | TSPAN17 | 0 | 0 | 0 | 0 | 1 | 0 | 1 |
| FOXJ2 | TMEM257 | 0 | 0 | 0 | 0 | 1 | 0 | 1 |
| EN1 | PHF11 | 0 | 0 | 1 | 0 | 0 | 0 | 1 |
| FOXJ2 | HOXA10 | 0 | 0 | 0 | 0 | 1 | 0 | 1 |
| EN1 | FDXR | 0 | 0 | 1 | 0 | 0 | 0 | 1 |
| EN1 | SQSTM1 | 0 | 0 | 1 | 0 | 0 | 0 | 1 |
| FOXJ2 | MXI1 | 0 | 0 | 0 | 0 | 1 | 0 | 1 |
| FOXJ2 | ANAPC11 | 0 | 0 | 0 | 0 | 1 | 0 | 1 |
| EN1 | MIR122 | 0 | 0 | 1 | 0 | 0 | 0 | 1 |
| FOXJ2 | AMBN | 0 | 0 | 0 | 0 | 1 | 0 | 1 |
| EN1 | CNTFR | 0 | 0 | 1 | 0 | 0 | 0 | 1 |
| EN1 | EMILIN1 | 0 | 0 | 1 | 0 | 0 | 0 | 1 |
| FOXJ2 | ZBTB20 | 0 | 0 | 0 | 0 | 1 | 0 | 1 |
| EN1 | CLC | 0 | 0 | 0 | 0 | 1 | 0 | 1 |
| FOXJ2 | SCG3 | 0 | 0 | 0 | 0 | 1 | 0 | 1 |
| EN1 | CTCF | 0 | 0 | 0 | 0 | 1 | 0 | 1 |
| FOXJ2 | PCF11 | 0 | 0 | 0 | 0 | 1 | 0 | 1 |
| EN1 | THPO | 0 | 0 | 1 | 0 | 0 | 0 | 1 |
| EN1 | GAP43 | 0 | 0 | 0 | 0 | 1 | 0 | 1 |
| EN1 | IFI30 | 0 | 0 | 1 | 0 | 0 | 0 | 1 |
| EN1 | DMBX1 | 0 | 0 | 1 | 0 | 0 | 0 | 1 |
| EN1 | SYNPO | 0 | 0 | 1 | 0 | 0 | 0 | 1 |
| EN1 | SMAD2 | 0 | 0 | 1 | 0 | 0 | 0 | 1 |
| SATB1 | CD8B | 0 | 0 | 0 | 0 | 0 | 1 | 1 |
| EN1 | OR8G5 | 0 | 0 | 1 | 0 | 0 | 0 | 1 |
| EN1 | NCOA7 | 0 | 0 | 1 | 0 | 0 | 0 | 1 |
| FOXJ2 | GSTO2 | 0 | 0 | 0 | 0 | 1 | 0 | 1 |
| FOXJ2 | TEAD1 | 0 | 0 | 0 | 0 | 1 | 0 | 1 |
| EN1 | ABCC8 | 0 | 0 | 1 | 0 | 0 | 0 | 1 |
| EN1 | PIK3R2 | 0 | 0 | 1 | 0 | 0 | 0 | 1 |
| EN1 | FBXO8 | 0 | 0 | 1 | 0 | 0 | 0 | 1 |
| EN1 | GNRH1 | 0 | 0 | 0 | 0 | 1 | 0 | 1 |
| EN1 | MEOX1 | 0 | 0 | 1 | 0 | 0 | 0 | 1 |
| EN1 | SNCAIP | 0 | 0 | 1 | 0 | 0 | 0 | 1 |
| EN1 | RNASEH2C | 0 | 0 | 1 | 0 | 0 | 0 | 1 |
| EN1 | NRG4 | 0 | 0 | 1 | 0 | 0 | 0 | 1 |
| FOXJ2 | SARAF | 0 | 0 | 0 | 0 | 1 | 0 | 1 |
| FOXJ2 | FOXN1 | 0 | 0 | 0 | 0 | 1 | 0 | 1 |
| FOXJ2 | CD68 | 0 | 0 | 0 | 0 | 1 | 0 | 1 |
| FOXJ2 | POGZ | 0 | 0 | 0 | 0 | 1 | 0 | 1 |
| EN1 | ELL | 0 | 0 | 1 | 0 | 0 | 0 | 1 |
| FOXJ2 | NDUFB5 | 0 | 0 | 0 | 0 | 1 | 0 | 1 |
| EN1 | NSRP1 | 0 | 0 | 1 | 0 | 0 | 0 | 1 |
| FOXJ2 | CHN2 | 0 | 0 | 0 | 0 | 1 | 0 | 1 |
| EN1 | SARM1 | 0 | 0 | 1 | 0 | 0 | 0 | 1 |
| FOXJ2 | LRRTM3 | 0 | 0 | 0 | 0 | 1 | 0 | 1 |
| EN1 | C19orf52 | 0 | 0 | 1 | 0 | 0 | 0 | 1 |
| EN1 | KRTAP3-3 | 0 | 0 | 1 | 0 | 0 | 0 | 1 |
| FOXJ2 | PRR34 | 0 | 0 | 0 | 0 | 1 | 0 | 1 |
| FOXJ2 | TMEM229B | 0 | 0 | 0 | 0 | 1 | 0 | 1 |
| FOXJ2 | SRSF7 | 0 | 0 | 0 | 0 | 1 | 0 | 1 |
| EN1 | NEO1 | 0 | 0 | 0 | 0 | 1 | 0 | 1 |
| EN1 | NLRC3 | 0 | 0 | 1 | 0 | 0 | 0 | 1 |
| EN1 | PRDX6 | 0 | 0 | 1 | 0 | 0 | 0 | 1 |
| EN1 | MCF2 | 0 | 0 | 1 | 0 | 0 | 0 | 1 |
| FOXJ2 | CYP26A1 | 0 | 0 | 0 | 0 | 1 | 0 | 1 |
| EN1 | PTPN12 | 0 | 0 | 1 | 0 | 0 | 0 | 1 |
| EN1 | PHKA1 | 0 | 0 | 1 | 0 | 0 | 0 | 1 |
| FOXJ2 | CALCOCO1 | 0 | 0 | 0 | 0 | 1 | 0 | 1 |
| FOXJ2 | PRDM1 | 0 | 0 | 0 | 0 | 1 | 0 | 1 |
| EN1 | TMEM59L | 0 | 0 | 0 | 0 | 1 | 0 | 1 |
| EN1 | RHOQ | 0 | 0 | 1 | 0 | 0 | 0 | 1 |
| FOXJ2 | SKIDA1 | 0 | 0 | 0 | 0 | 1 | 0 | 1 |
| EN1 | LRRN3 | 0 | 0 | 1 | 0 | 0 | 0 | 1 |
| EN1 | OSBP | 0 | 0 | 1 | 0 | 0 | 0 | 1 |
| FOXJ2 | FABP4 | 0 | 0 | 0 | 0 | 1 | 0 | 1 |
| EN1 | MED16 | 0 | 0 | 1 | 0 | 0 | 0 | 1 |
| EN1 | TBX21 | 0 | 0 | 1 | 0 | 0 | 0 | 1 |
| FOXJ2 | PCDH17 | 0 | 0 | 0 | 0 | 1 | 0 | 1 |
| EN1 | FBXL6 | 0 | 0 | 1 | 0 | 0 | 0 | 1 |
| FOXJ2 | NFIX | 0 | 0 | 0 | 0 | 1 | 0 | 1 |
| FOXJ2 | MITF | 0 | 0 | 0 | 0 | 1 | 0 | 1 |
| FOXJ2 | OLR1 | 0 | 0 | 0 | 0 | 1 | 0 | 1 |
| EN1 | FAM53B | 0 | 0 | 0 | 0 | 1 | 0 | 1 |
| FOXJ2 | PHACTR3 | 0 | 0 | 0 | 0 | 1 | 0 | 1 |
| FOXJ2 | TASP1 | 0 | 0 | 0 | 0 | 1 | 0 | 1 |
| EN1 | CCNB1IP1 | 0 | 0 | 1 | 0 | 0 | 0 | 1 |
| EN1 | NPY5R | 0 | 0 | 1 | 0 | 0 | 0 | 1 |
| EN1 | VWCE | 0 | 0 | 1 | 0 | 0 | 0 | 1 |
| EN1 | MFN1 | 0 | 0 | 1 | 0 | 0 | 0 | 1 |
| EN1 | GTF2A1L | 0 | 0 | 1 | 0 | 0 | 0 | 1 |
| FOXJ2 | FSTL1 | 0 | 0 | 0 | 0 | 1 | 0 | 1 |
| EN1 | ASAP2 | 0 | 0 | 1 | 0 | 0 | 0 | 1 |
| EN1 | PER3 | 0 | 0 | 1 | 0 | 0 | 0 | 1 |
| EN1 | CAPN11 | 0 | 0 | 1 | 0 | 0 | 0 | 1 |
| EN1 | CEP128 | 0 | 0 | 1 | 0 | 0 | 0 | 1 |
| EN1 | CLDN20 | 0 | 0 | 1 | 0 | 0 | 0 | 1 |
| EN1 | APPL1 | 0 | 0 | 1 | 0 | 0 | 0 | 1 |
| EN1 | PIANP | 0 | 0 | 1 | 0 | 0 | 0 | 1 |
| EN1 | CASP9 | 0 | 0 | 1 | 0 | 0 | 0 | 1 |
| EN1 | WWTR1 | 0 | 0 | 1 | 0 | 0 | 0 | 1 |
| EN1 | HOXB8 | 0 | 0 | 0 | 0 | 1 | 0 | 1 |
| EN1 | KLF15 | 0 | 0 | 0 | 0 | 1 | 0 | 1 |
| FOXJ2 | C12ORF54 | 0 | 0 | 0 | 0 | 1 | 0 | 1 |
| EN1 | WDR35 | 0 | 0 | 1 | 0 | 0 | 0 | 1 |
| EN1 | POLRMT | 0 | 0 | 1 | 0 | 0 | 0 | 1 |
| EN1 | TMPRSS4 | 0 | 0 | 1 | 0 | 0 | 0 | 1 |
| EN1 | TAS2R4 | 0 | 0 | 1 | 0 | 0 | 0 | 1 |
